# Supplementary material for: Spatial tomography of light resolved in time, spectrum, and polarisation
Source: Nat Commun. 2022 Jul 25;13:4294. doi: 10.1038/s41467-022-31814-2 (PMC9314355; doi:10.1038/s41467-022-31814-2)
Supplement: Supplementary file 1 — Supplementary Information [file 41467_2022_31814_MOESM1_ESM.pdf]

Supplementary Information: Spatial tomography of light resolved in time, spectrum, and polarisation

Martin Plöschner,[1,*] Marcos Maestre Morote,[1] Daniel Dahl,[1] Mickael Mounaix,[1] Greta Light,[2] Aleksandar D. Rakić,[1] and Joel Carpenter[1]

[1]*School of Information Technology and Electrical Engineering, The University of Queensland, Brisbane, QLD 4072, Australia*

[2]*II-VI Incorporated, 48800 Milmont Dr., Fremont CA 94538, USA*

*Corresponding author; E-mail: m.ploschner@uq.edu.au

Supplementary Note 1: EXPERIMENTAL SETUP

The schematic of the experimental setup is in Fig. 1. Light from the 856nm VCSEL array (VDC-2183-055, 25G, 1x12, Finisar), controlled by the TOPAZ BOA evaluation board (Finisar), is collimated by an aspheric lens L_1 ($f_1 = 10\,\text{mm}$) and passed through a tandem of half ($\lambda/2$) and quarter-waveplate ($\lambda/4$) for polarisation adjustment of the VCSEL laser. The light is subsequently separated into two light-paths by a non-polarising beam-splitter (NPBS). The reflected optical path is used for beam monitoring purposes, such as verification of which VCSELs in the array are active. The VCSEL array is imaged onto a CCD (Thorlabs, DCC1645C-HO) via achromatic-doublet lens L_2 ($f_2 = 75\,\text{mm}$). The optical path that transmits through the NPBS is separated into horizontal and vertical polarisation by a polarising beam-splitter. A $\lambda/2$ waveplate in the right optical path is used to match the polarisation axis of the light to that of the spatial light modulator (SLM, Meadowlark, 1920x1152). Another $\lambda/2$ waveplate is used after the SLM in the left optical path to enable recombination of the orthogonal polarisations using a PBS. The light is then coupled into a single-mode fibre (SMF, Corning, HI780) using an achromatic lens L_3 ($f_3 = 25\,\text{mm}$).

The light is then split 99/1 by a coupler. The 1% arm is used to monitor the power coupled into SMF using a powermeter (Thorlabs, PM100USB). The 99% arm is further split 50/50, with one arm guiding light into an optical spectrum analyser (OSA, Agilent 86140B) and the other arm connected to a high-speed detector (New Focus, Model 1484-A-50, 22 GHz bandwidth). We monitor the DC bias of the detector with an NI DAQ card (USB-6002). Monitoring the DC bias is crucial as the DC component is filtered out by a DC block before we amplify the signal

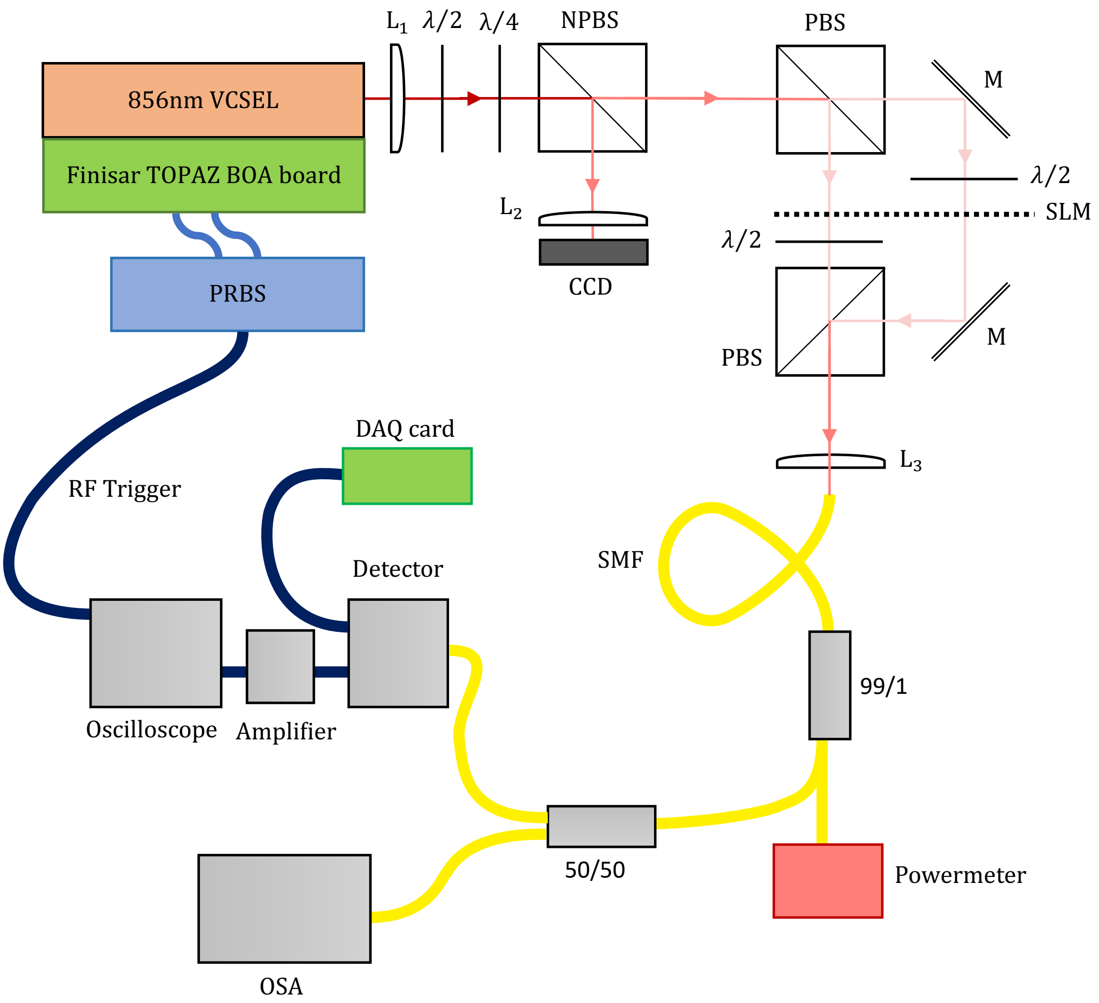

Supplementary Figure 1 **Experimental setup** The light source can be independently analysed in H and V polarisation using the spatial light modulator, the oscilloscope and the optical spectrum analyser (OSA). L_1 - lens, $f_1 = 10\,\text{mm}$; $\lambda/2$ - half-waveplate; $\lambda/4$ - quarter-waveplate; NPBS - non-polarising beam-splitter; L_2 - lens, $f_2 = 75\,\text{mm}$, CCD - camera; PBS - polarising beam-splitter; SLM - spatial light modulator; L_3 - lens, $f_3 = 25\,\text{mm}$; SMF - single-mode fibre; PRBS - pseudo-random binary sequence generator.

with an amplifier (Picosecond 5840B, 20.8 dB gain at 10 GHz, bandwidth 80 kHz to 13.5 GHz) and send it to the oscilloscope (Agilent, 86100A, module Agilent 86116A, 63 GHz electrical maximum sampling rate). As a result, only the AC signal component is collected by the oscilloscope and the DC component has to be added numerically in the post-processing of the signal. This step is crucial since the projective measurements are intensity-based and therefore we need to know both the AC and the DC signal components to fully reconstruct the density matrix of the input light. Given the critical nature of the DC bias value for reconstruction, we compare the value measured by the NI DAQ card with the power measured by the powermeter, accounting for coupler split ratio, amplifier gain and the detector parameters to convert voltage into the detected power.

The acquisition of the oscilloscope traces is synchronised with the VCSEL modulation via pseudorandom binary sequence (PRBS) generator (Agilent, N4962A). PRBS provides a radio-frequency (RF) signal that drives the VCSEL at 10 GHz via TOPAZ BOA board with an amplitude of 0.7 V. We use the differential signal scheme for modulation, using both output ports of the PRBS to limit the modulation signal noise. Each time the PRBS sends the sequence for modulation, it triggers the oscilloscope for the acquisition of temporal traces. The delay between the PRBS signal and the collected VCSEL signal is found by maximising the cross-correlation between the two signals. Sometimes this leads to plotting of temporal traces where the VCSEL appears to react before the driving signal. However, this is only an artefact of trying to align the driving signal and the response for plotting purposes.

Supplementary Note 2: SYSTEM ALIGNMENT, CALIBRATION PROCEDURES AND DATA PROCESSING

A. System alignment and calibrations

For optimal operation of the system (Supplementary Fig. 1), we first need to remove the optical aberrations. The procedure to remove aberrations is as follows.

We substitute the VCSEL source with a laser diode (Thorlabs, LP852-SF30) coupled into a single-mode fibre that is identical to the SMF used on the detection site during Stokes projective measurements. We collimate the beam coming from the source SMF and manually approximately maximise the coupling into SMF on the detection site. Afterwards, we employ a Nelder-Mead optimisation method that uses a combination of Zernike polynomials up to order six to correct for system aberrations to optimise the coupling into the detection SMF. Nelder-Mead is a multidimensional space optimisation algorithm which in our case optimises a function of power coupled into SMF. This function depends on the magnitude of Zernike aberration terms such as tilt, defocus, coma, trefoil, as well as SLM mask centre offsets in x and y that are applied to co-align the beam with the SLM masks. The whole process is performed independently for both H and V polarisations which enables the determination of aberration terms (due to lenses and the SLM in the system) and mask centres for optical paths at each polarisation. We note that, in principle, the VCSEL diode itself could be used for this alignment step. This could be done by using the OSA as a detector with spectral bandwidth set to only cover the fundamental mode of the VCSEL. However, we found the power in the fundamental mode of the VCSEL diode too low and unstable for the determination of the higher-order aberrations with high fidelity. Only tilt and defocus terms could be determined with high enough precision directly with the VCSEL diode. Additionally, there can potentially be additional aberration of the fundamental mode of the VCSEL due to carrier injection patterns and asymmetries of the laser cavity. Applying a Nelder-Mead on the aberrated source would bias the results. It is therefore better to chose laser diode coupled into an SMF as a source as in this case the source has virtually no higher-order aberration terms.

After the determination of the higher-order aberrations, we remove the source SMF with light from the Thorlabs diode and position the VCSEL diode such that it is as close as possible to where the source SMF facet was. The higher-order aberrations remain practically unchanged after this step. The only Zernike terms that require adjustment are the tilt and the defocus, which we find by maximising the fundamental mode peak on the OSA using the same Nelder-Mead method as in the first step. We subsequently verify the fundamental mode profile by raster scanning the fundamental mode over the detection SMF. The raster scan is performed by adding tilt to the SLM mask and detecting the power in the fundamental peak using the OSA for each tilt value.

In the next step, we aim to again determine the relative transverse offset of the mask and the VCSEL beam on the SLM. Ideally, the beam should be illuminating the centre of the mask but after swapping the lasers this is generally no longer the case. To find the centre of the mask relative to the beam with a precision up to a single pixel of the SLM, we perform a π-flip horizontal/vertical scan in the vicinity of the mask centre and measure the intensity detected by the SMF. The π flip converts the fundamental beam into an LP11 beam when the π step occurs in the centre of the beam. Therefore, the x, y coordinates of the π step where we detect the minimum intensity coupled into an SMF correspond to the position of the beam on the mask. With the relative offset of the mask and the beam established, we move the SLM mask to the centre of the beam. The whole procedure is repeated for both H and V polarisation. If the detected mask offset is large, we perform the tilt, defocus and the mask offset step again until the values start

converging for all tilt, defocus and mask offsets. We verify the centering by displaying a spiral phase mask on the SLM, which converts the fundamental mode into an LG01. In an ideally aligned system, the SLM raster scan with a spiral phase mask and with the OSA bandwidth setup to only detect power in the fundamental mode of the VCSEL, results in a detected beam profile with symmetric LG01 and high contrast between the singularity and the ring. We use the contrast as a metric to fine-tune the x and y position of the SLM mask using Nelder-Mead algorithm.

B. Spatio-spectral data processing

The spectrum of the VCSEL system is sensitive to small temperature variations of the VCSEL and the TOPAZ BOA board does not allow for temperature control. We have observed the temperature changes of around $0.3°$ during the course of the series of projective measurements, which typically led to 30 pm spectrum offset. We tracked the peak position of the fundamental peak with respect to its initial spectral position and numerically shifted the collected spectra based on this offset. This procedure ensures that the intensity in a given wavelength bin always corresponds to the same spectral peak. This approach only works for small temperature changes when the spectrum remains the same and just shifts to either longer or shorter wavelengths. For bigger temperature changes, the shape of the spectrum itself changes and the method cannot be applied.

The spatially integrated power over all the pixels of the SLM raster scan has to account for a factor that arises due to overlay of the detection regions. For example, very fine tilt-step on the SLM produces small lateral offset of the beam in the detection SMF plane. In fact, the step can be smaller than the core of the SMF which means that a large portion of the already detected power for the previous tilt value is detected again. We calibrate for the power multiplicative factor for each lateral sampling rate that we use by measuring the total power in the beam using an auxiliary powermeter with a large core 200 μm MMF fibre that captures all the light at once. With the factor included, the integrated spatial power in the OSA spectrum is the same as the power detected by the MMF fibre.

C. Spatio-temporal data processing

The oscilloscope traces were averaged over 80 measurements for each projective SLM mask. We subsequently applied the lowpass Butterworth filter of order 5 with the cutoff frequency equal to half the sampling frequency 63 GHz. The DC signal component that is DC blocked before the amplifier was numerically added to the oscilloscope trace based on the monitoring of the DC bias port of the detector. The DC component magnitude was cross-verified with a power measured by a powermeter.

The spatially integrated temporal response of VCSEL is calculated in an equivalent way to the Section B above. The multiplication factors for different lateral sampling rates remain unchanged as we use the identical SMF for detection.

Supplementary Note 3: THEORY OF THE HIGH-DIMENSIONAL STOKES ANALYSIS

In this section, we aim to explain the basic principles of the high-dimensional Stokes method. The core mechanism needed to reconstruct the unknown state of light is a series of projective intensity measurements performed in an arbitrary basis. This section provides mathematical formalism to determine the projective states in a user-selected basis of interest and shows how the intensity measurements translate into the knowledge of the Stokes vector and density matrix of the unknown state. We use quantum formalism throughout as this simplifies the notation and makes the material more accessible.

A mixture of N pure states $|\psi_i\rangle$ (in our case spatial modes) can be represented by a density matrix $\hat{\rho}$ as

$$\hat{\rho} = \sum_{i=1}^{N} p_i |\psi_i\rangle\langle\psi_i|, \tag{1}$$

where p_i is the probability of state $|\psi_i\rangle$ in the mixture. The density matrix is a positive semi-definite, Hermitian matrix with trace one. We assume that all the states in the mixture are orthogonal with respect to each other ($\langle\psi_i|\psi_j\rangle = \delta_{ij}$), which ensures the uniqueness of the density matrix. When the states in the mixture are not orthogonal, the density matrix can still be constructed, but it is no longer unique, which in turn means that the knowledge of the matrix does not contain unique information about the states and their probabilities in the mixture.

The Pauli matrices ($\hat{\sigma}_i$), together with the identity matrix ($\hat{\sigma}_0$), form an orthogonal basis for a space of 2×2 Hermitian matrices. That implies that any 2×2 Hermitian matrix can be expressed as a linear combination of Pauli

matrices and the identity matrix. Since the density matrix is a Hermitian matrix, it can be expressed using the Pauli matrices as

$$\hat{\rho} = \frac{1}{2}\sum_{i=0}^{3} S_i \hat{\sigma}_i, \tag{2}$$

where S_i are equivalent to the vector elements of the Stokes vector, or, more generally, the expectation values of each Pauli matrix $\hat{\sigma}_i$. Therefore, to determine the density matrix of a given mixture of states, we need to find the expectation values S_i for each Pauli matrix. We note that the factor of 1/2 in front of the sum appears because the Pauli matrices need to be normalised by finding the Frobenius norm of a matrix:

$$||\hat{A}||_F = \left(\sum_{i,j} |A_{i,j}|^2\right)^{\frac{1}{2}}, \tag{3}$$

where $\hat{A}$ is the matrix to be normalised. Each Pauli matrix is divided by the square of the norm. If the factor of 1/2 is omitted, the probabilities of the states in the mixture would be doubled and the sum of the probabilities would be equal to 2, not 1. While the normalisation seems like a minor issue that can be readily fixed *a posteriori* based on the non-physical nature of the probability sum of all states being 2, the normalisation is of immense importance for higher-dimensional case, where each matrix has a different normalisation factor and its omission is not easy to track down, with the density matrix making non-sensical predictions.

The expectation value of the Pauli matrix $\hat{\sigma}_i$ for a mixture given by the density matrix $\hat{\rho}$ is given by:

$$\langle\hat{\sigma}_i\rangle_{\hat{\rho}} = \mathrm{Tr}\left(\hat{\rho}\hat{\sigma}_i\right) = \frac{1}{2}\mathrm{Tr}\left(\sum_{j=0}^{3} S_j\hat{\sigma}_j\hat{\sigma}_i\right) = \frac{1}{2}\sum_{j=0}^{3} S_j \mathrm{Tr}\left(\hat{\sigma}_j\hat{\sigma}_i\right) = \sum_{j=0}^{3} S_j\delta_{ij} = S_i \tag{4}$$

To measure the expectation values S_i, it is useful to express each Pauli matrix using its eigenvalues and eigenvectors. The eigenvectors can be evaluated on an arbitrary orthogonal basis, depending on the physical characteristics of the system that we want to probe. Here we choose the polarisation basis for demonstration purposes, but we can equivalently express the eigenvectors in a spatial orthogonal basis as we do in Figure 2(b) in the main manuscript. The Pauli matrices can be expressed in the polarisation basis using the eigenvectors and eigenvalues (κ) as follows:

**Pauli matrix**	**eigenvalues**	**eigenvectors**	**matrix decomposition**	
$\hat{\sigma}_0 = \begin{pmatrix}1 & 0\\ 0 & 1\end{pmatrix}$	$\kappa_0^0 = 1$<br>$\kappa_0^1 = 1$	$\begin{pmatrix}1\\0\end{pmatrix} := \lvert H\rangle$<br>$\begin{pmatrix}0\\1\end{pmatrix} := \lvert V\rangle$	$\rightarrow \quad \hat{\sigma}_0 = \kappa_0^0\lvert H\rangle\langle H\rvert + \kappa_0^1\lvert V\rangle\langle V\rvert$	(5)
$\hat{\sigma}_1 = \begin{pmatrix}0 & 1\\ 1 & 0\end{pmatrix}$	$\kappa_1^0 = 1$<br>$\kappa_1^1 = -1$	$\frac{1}{\sqrt{2}}\begin{pmatrix}1\\1\end{pmatrix} := \lvert D\rangle$<br>$\frac{1}{\sqrt{2}}\begin{pmatrix}1\\-1\end{pmatrix} := \lvert A\rangle$	$\rightarrow \quad \hat{\sigma}_1 = \kappa_1^0\lvert D\rangle\langle D\rvert + \kappa_1^1\lvert A\rangle\langle A\rvert$	(6)
$\hat{\sigma}_2 = \begin{pmatrix}0 & -i\\ i & 0\end{pmatrix}$	$\kappa_2^0 = 1$<br>$\kappa_2^1 = -1$	$\frac{1}{\sqrt{2}}\begin{pmatrix}1\\i\end{pmatrix} := \lvert R\rangle$<br>$\frac{1}{\sqrt{2}}\begin{pmatrix}1\\-i\end{pmatrix} := \lvert L\rangle$	$\rightarrow \quad \hat{\sigma}_2 = \kappa_2^0\lvert R\rangle\langle R\rvert + \kappa_2^1\lvert L\rangle\langle L\rvert$	(7)
$\hat{\sigma}_3 = \begin{pmatrix}1 & 0\\ 0 & -1\end{pmatrix}$	$\kappa_3^0 = 1$<br>$\kappa_3^1 = -1$	$\begin{pmatrix}1\\0\end{pmatrix} := \lvert H\rangle$<br>$\begin{pmatrix}0\\1\end{pmatrix} := \lvert V\rangle$	$\rightarrow \quad \hat{\sigma}_3 = \kappa_3^0\lvert H\rangle\langle H\rvert + \kappa_3^1\lvert V\rangle\langle V\rvert$	(8)

Substituting the expressions for the Pauli matrices (Eqs. (5-8)) to the equation for the expectation values (Eq. 4) leads to the following set of equations that constitute the set of projective measurements that need to be performed in order to obtain the expectation values S_i:

$$S_0 = \mathrm{Tr}\,(\hat{\rho}\hat{\sigma}_0) = \kappa_0^0\langle H|\hat{\rho}|H\rangle + \kappa_0^1\langle V|\hat{\rho}|V\rangle \tag{9}$$

$$S_1 = \mathrm{Tr}\,(\hat{\rho}\hat{\sigma}_1) = \kappa_1^0\langle D|\hat{\rho}|D\rangle + \kappa_1^1\langle A|\hat{\rho}|A\rangle \tag{10}$$

$$S_2 = \mathrm{Tr}\,(\hat{\rho}\hat{\sigma}_2) = \kappa_2^0\langle R|\hat{\rho}|R\rangle + \kappa_2^1\langle L|\hat{\rho}|L\rangle \tag{11}$$

$$S_3 = \mathrm{Tr}\,(\hat{\rho}\hat{\sigma}_3) = \kappa_3^0\langle H|\hat{\rho}|H\rangle + \kappa_3^1\langle V|\hat{\rho}|V\rangle. \tag{12}$$

The eigenvalues (κ) are fully determined by Pauli matrices and do not change if the polarisation basis is swapped to spatial basis or any other physical basis of interest. The S_i values are only affected by the state of the explored system, given by $\hat{\rho}$, in the basis of interest.

A total of six projective measurements ($\langle H|\hat{\rho}|H\rangle, \langle V|\hat{\rho}|V\rangle, \langle D|\hat{\rho}|D\rangle, \langle A|\hat{\rho}|A\rangle, \langle R|\hat{\rho}|R\rangle, \langle L|\hat{\rho}|L\rangle$) is needed to obtain all expectation values and thus fully reconstruct the density matrix of the mixture using Eq. 2. The eigenvalues of the obtained density matrix correspond to the probabilities and the eigenvectors to the states in the mixture. We note that the elements of eigenvectors correspond to the linear combination coefficients in the original physical basis, in this case, the polarisation basis ($|H\rangle, |V\rangle$). Again, the projective measurement can be performed in any physical basis of interest. For all experiments in our manuscript, we choose a basis of random orthogonal spatial modes. Despite the random, speckle like spatial projection states as depicted in Figure 2 in the main manuscript, the eigenvectors obtained from the density matrix combine the random projective states in a way that yields the well recognisable LP modes of the laser cavity. The added benefit of using random spatial modes is the equal sensitivity of the projective states to the spatial aspects of the original beam, making it more robust with respect to noise in scenarios where only one or two LP spatial modes are present in the source beam. In such a case, most of the projective measurements performed in the LP spatial basis would just contribute to noise as there is nothing these projective states can detect if the spatial aspects they are sensitive to are not present. The key message here is that the mathematical formalism presented is valid for any physical basis of interest and the basis can be chosen to match the physical system investigated.

Our example that uses Pauli matrices only allows for probing of the system with 2 states in the mixture. As mentioned, the Pauli matrices, together with the identity matrix form a basis for all 2×2 Hermitian matrices. The Pauli matrices span the Lie algebra of the SU(2) group, with each Pauli matrix corresponding to rotations. In simplified terms, by applying the Pauli matrices we rotate the unknown mixed state (object) in such a way that we can take the projective measurement (the shadow of the object from a certain angle). Rotating the object multiple times and looking at the shadow helps us to reconstruct the original object shape[1]. Knowing the expectation value for a certain Pauli matrix (certain rotation) is what tells us what the 'shadow' of the unknown mixed state looks like for a certain rotation. The full set of expectation values S_i then allows reconstruction of the density matrix. Any density matrix can be represented as a point on or inside a Bloch sphere. The points on the surface of the Bloch sphere represent the pure states whereas the points inside the Bloch sphere represent the mixed states. The origin of the sphere represents a completely mixed state where the probabilities of the states are 50%/50%. This corresponds to unpolarised light if we are measuring the polarisation state of light. At the origin of the Bloch sphere, it is not possible to determine the states in the mixture. The 50%/50% can correspond to any mixture of the polarisation states. Similarly, it is not possible to determine the spatial eigenstates if the density matrix corresponds to the origin of the Bloch sphere. The 50%/50% can correspond to any mixture of spatial states.

Similar rotations can be defined in higher dimensions. Gell-Mann matrices span the Lie algebra of the SU(3) group[2]. The Gell-Mann matrices are the 3-dimensional analogues of Pauli matrices, and together with the identity matrix, the Gell-Mann matrices form the basis for all 3×3 Hermitian matrices. We can again find the expectation values S_i for each Gell-Mann matrix, by finding the eigenvalues and eigenvectors of each Gell-Mann matrix and expressing each Gell-Mann matrix similar to Eqs. (5-8). Inserting the Gell-Mann matrices expressed in such a way into (Eq. 4), we obtain 9 equations for the expectation values S_i. We need a total of 15 projective measurements to obtain the expectation values S_i, where S_i can be seen as a high-dimensional analogue of the Stokes vector.

Finally, for the analysis of an arbitrary N dimensional case, we can use the generalised Gell-Mann matrices that span the Lie algebra of the SU(N) group. Together with the identity matrix, the generalised Gell-Mann matrices form a basis for $N \times N$ Hermitian matrices. We need to know N^2 expectation values S_i to fully reconstruct the density matrix, which requires $2N^2 - N$ projective measurements. We reiterate that each generalised Gell-Mann matrix has to be normalised using Eq. (3), otherwise, the reconstructed density matrix leads to non-physical results. Similar to the two-dimensional case, the reconstructed density matrix can be represented by a point on a Bloch hyperball, with points on the surface corresponding to the pure states and the points inside corresponding to the mixed state. Whenever, the probabilities of the two states match, it is impossible to determine the spatial profiles of these two states. This can be seen in our temporal data in Figure 4 of the main manuscript and in the corresponding Supplementary Media.

In the immediate vicinity of the crossing-points of probability curves for each state, the spatial profiles of the states that cross each other become undefined.

In principle, it is possible to reduce the number of measurements to N^2, which is the number of expectation values S_i we need to know to reconstruct the density matrix. This can be understood in the simple polarisation case scenario by realising that the $|D\rangle$ and $|A\rangle$ projections are directly related to $|H\rangle$ and $|V\rangle$ measurements ($|D\rangle = 1/\sqrt{2}\,(|H\rangle + |V\rangle)$, $|A\rangle = 1/\sqrt{2}\,(|H\rangle - |V\rangle)$), and indeed in many Stokes polarimetry applications only $N^2 = 4$ measurements are required for $N = 2$ polarisation space, instead of the $2N^2 - N = 6$. While the reduced number of measurements increases the speed at which we measure the density matrix, the inclusion of the redundant measurements decreases the noise and additionally makes the method much simpler to implement programmatically for the high-dimensional case scenario. Interestingly, when the measurements are not performed sequentially, as in our case, but in parallel as in the case of multiplane-light-converter[3,4], it is not even physically possible to route the light losslessly through the optical system with only N^2 output ports. The nature of the measurement forces $2N^2 - N$ output ports.

In summary, all we need to reconstruct the density matrix is a series of projective measurements of the form:

$$\langle\psi|\hat{\rho}|\psi\rangle. \tag{13}$$

The procedure to perform such measurements using the spatial mode basis is elaborated in Supplementary Note 4. These measurements are subject to noise, which sometimes results in the reconstructed density matrix that is not positive, semi-definite, *i.e.* nonphysical negative probabilities predicted. As such, this matrix cannot directly represent a physical mixture. We numerically find the nearest positive, semidefinite matrix by using the procedure described by Higham[5]. This modifies both the detected eigenvalues and eigenvectors. The larger the measurement noise, the further away our measured matrix is from the nearest positive, semidefinite matrix, and the larger the distortion to measured eigenvalues and eigenstates. The effect of noise is explored and simulated in Supplementary Note 9.

Supplementary Note 4: EXPERIMENTAL MEASUREMENT OF FIELD OVERLAPS

Supplementary Figure 2 shows how the expectation values of the type presented in Eq. 13 are measured in our experimental setup. Imagine a hypothetical light coming from the detection SMF as illustrated in Supplementary Figure 2 (a). Since the fibre is single-mode, the light has to have a Gaussian profile. The light is collimated with a lens L1 and passes through the SLM. The mask displayed on the SLM converts the incoming light in a way that generates a hypothetical LP11 profile at the source plane. The source can be an arbitrary light source. So far, all the light launched into the system was purely hypothetical, albeit equivalent to what would physically happen if the SMF was used as a light source.

Now imagine the real experimental scenario with physical light coming from the source (Supplementary Figure 2 (b)). For illustrative purposes, let us assume that the source is in a pure Gaussian beam state, but in reality, the source density matrix can be any mixture of orthogonal spatial states. The Gaussian beam propagates from the source, passes through the SLM mask and is converted to an LP11 mode in the SMF plane. The power coupled into the SMF is proportional to the square of the overlap integral ($\left|\int E_1 E_2^* \,\mathrm{d}A\right|^2$) between the hypothetical field that comes out of the SMF (E_1) and the field from the source modified by the SLM mask (E_2). Crucially, the square of the overlap integral is conserved everywhere within the system due to light reciprocity as illustrated in Supplementary Figure 2 (c) - with the conservation of the square of the field overlap not just in the source and the detection plane, but in any plane normal to the optical axis in between the two extremities. That means that the overlap in the SMF plane has the same value as the overlap in the source plane, where we perform the projective measurements. In a nutshell, we hypothetically create the projective spatial modes in the source plane using the appropriate SLM masks. Physically, the projective measurement cannot be performed in the source plane because there is no physical light coming from the SMF. However, due to the conservation of square of the field overlap, the same projection value can be experimentally measured in the detection SMF plane of the system.

For the above to work optimally, we need to remove aberrations in the system (procedure described in Supplementary Note 2). Additionally, the number of total projective measurements (the spatial analyser states) can be greatly reduced if the projected spatial modes in the source plane have the same waist as the waist of the spatial modes in the mixture of the source. We estimate the waist of the spatial modes coming from the VCSEL by SLM raster-scanning and OSA filtering the fundamental mode. In cases, when the waist of the modes coming from the source cannot be determined, the spatial analyser state number has to be increased until all spatial aspects in the source can be represented by the measurement basis. In most of the scenarios, however, a judicious estimate of the source waist can be made, which allows matching the projective mode waist in the source plane.

The SLM masks generating the projective states in the source plane are calculated on the fly using a GPU. The algorithm is based on a modified Gerchberg-Saxton algorithm[6]. The illumination of the SLM from the hypothetical SMF is accounted for during the iterations between the source and the SLM plane, as well as the scaling by the lens

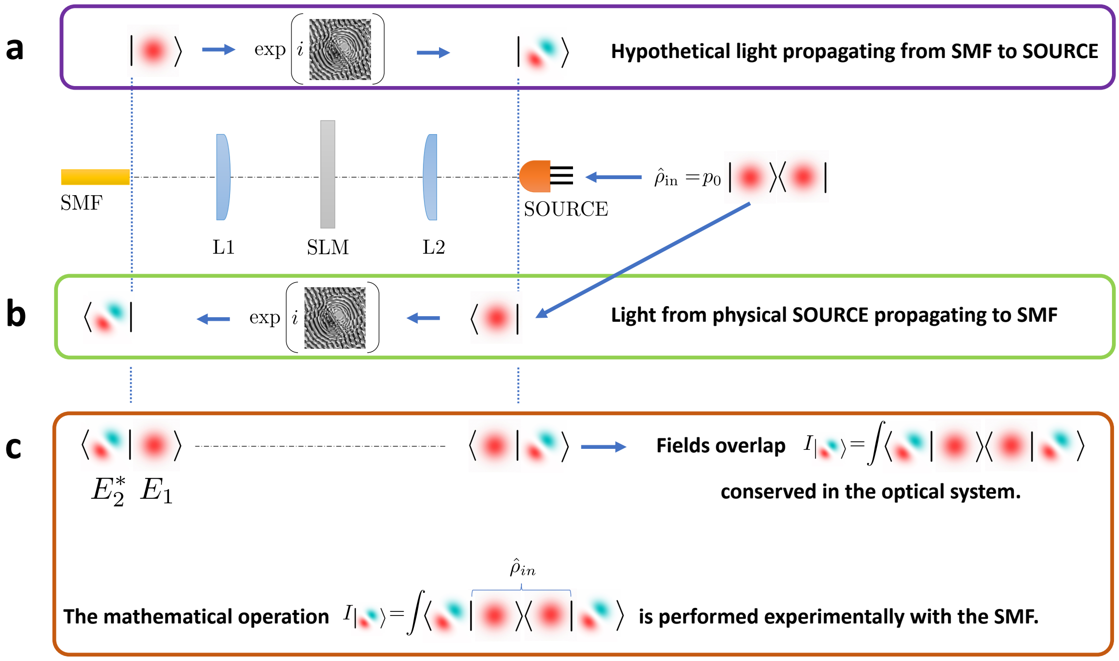

Supplementary Figure 2 **The conservation of fields overlap in the system and performing the fields overlap experimentally** The reciprocity of light propagation leads to the conservation of field overlap in the system. This means that the intensity (fields overlap) measured by the SMF is equivalent to the fields overlap at the source plane. The SMF measured intensity is equivalent to the expectation value as in Eq. 13 for a projective, spatial analyser state generated by the SLM mask. A series of projective SLM masks allows the reconstruction of the density matrix of the source. E_1 is the field of the fundamental mode of the SMF fibre, while E_2 is the LP11 field.

L2. Accounting for the Guassian-like illumination of the SLM from the SMF, completely eliminates the presence of the unwanted Gaussian envelope for all of the projections - removing a common problem that is often encountered in spatial tomography[7]. The algorithm applies a circular buffer zone around the target field which prevents unwanted dumping of the light by the algorithm into the target zone. The stop condition for the overlap between the target field and the obtained field is set to 0.99 which typically leads to 40% efficiency of the SLM mask. Better field overlap can be achieved at the cost of lower efficiency and a lower overall detected intensity, which makes the system more susceptible to measurement noise. We found the stop condition for overlap above 0.99 to be detrimental for the fidelity of the reconstructed states, likely due to increased measurement noise at low light levels of low-efficiency SLM masks.

We note that the mask efficiencies have to be accounted for during the projective measurements. Each projective measurement has a slightly different efficiency (variation typically around 10%). If the mask efficiency is not factored in, the intensity measurement for a given projective state is affected, which impacts the reconstructed density matrix.

Supplementary Note 5: SPATIO-SPECTRAL ANALYSIS (VERTICAL V POLARISATION)

Supplementary Figure 3 shows the spatio-spectral analysis of the VCSEL for the vertical V polarisation. Compared to the horizontal H polarisation case presented in Fig. 3 of the main manuscript, the power of LP11b spatial mode now dominates over LP11a mode, the opposite of the H polarisation case. The same effect can be observed for LP21a

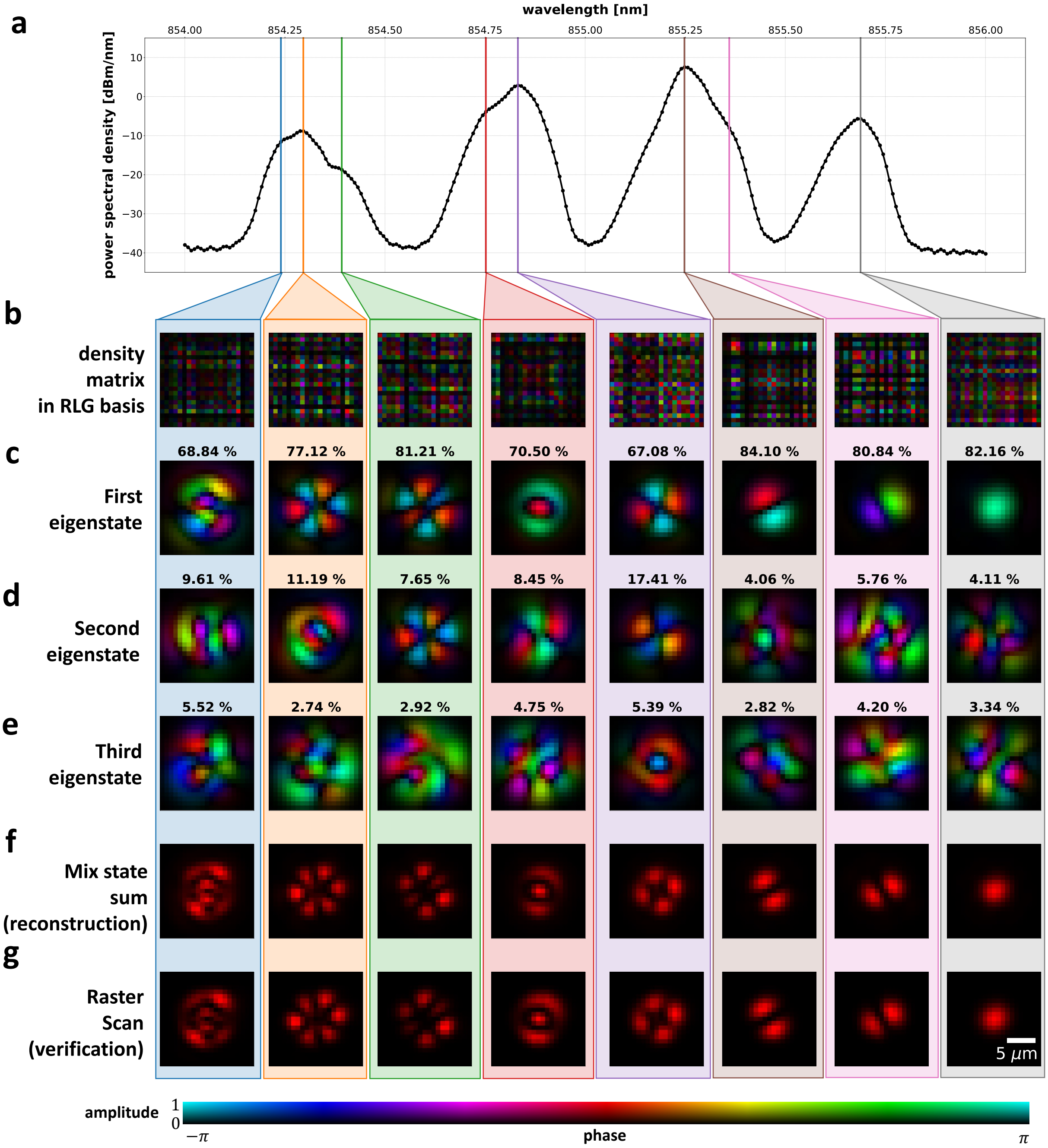

Supplementary Figure 3 **Spatio-spectral analysis of VCSEL (V polarisation)** (**a**) The spectrum has multiple spectral peaks, each corresponding to one or more spatial modes. (**b**) The measured density matrix, expressed in RLG basis, for colour-coded spectral peaks. (**c-e**) The first three most dominant spatial eigenstates and their corresponding probabilities as reconstructed from the density matrix (**b**) using the RLG basis. (**f**) The probability-weighted intensity sum of all reconstructed eigenstates obtained via state tomography perfectly matches the intensity profiles (**g**) obtained by raster scanning the VCSEL beam over SMF by adding tilt on the SLM and collecting the spectrum for each tilt value. The measured density matrix along with the reconstructed spatial eigenstates for each wavelength can be seen in Supplementary Media 2.

and LP21b. Some new modes can also be observed for V polarisation, including LP12 and even LP22. The access to modal distribution of the VCSEL for both H and V polarisation can be utilised for studies of mode-competition within the cavity, which have applications for engineering VCSELs with higher optical power, controlled intensity profiles and faster temporal dynamics.

Supplementary Note 6: SPATIO-TEMPORAL ANALYSIS (VERTICAL V POLARISATION)

Supplementary Figure 4 shows the temporal dynamics of the VCSEL over a 2 ns interval for the vertical V polarisation. The temporal snapshots are taken at identical times to H polarisation, meaning $t = 0.32$ ns and $t = 1.08$ ns respectively. In the H polarisation case, significant changes in modal probabilities as a function of time were observed during VCSEL modulation, especially for LP01 and LP21 modes that exhibited probability crossing between the low and high bias states. For V polarisation the dynamics is relatively stable, with only a marginal change in probability for LP11 mode. The observed temporal dynamics is in agreement with modally resolved light-current curves in Supplementary Figure 6 (a, c), with LP11 mode dominating over the whole bias modulation range and minimally affected in terms of power. Similarly, the LP21a and LP21b modes have relatively stable output power over the whole modulation range according to Supplementary Figure 6(a, c).

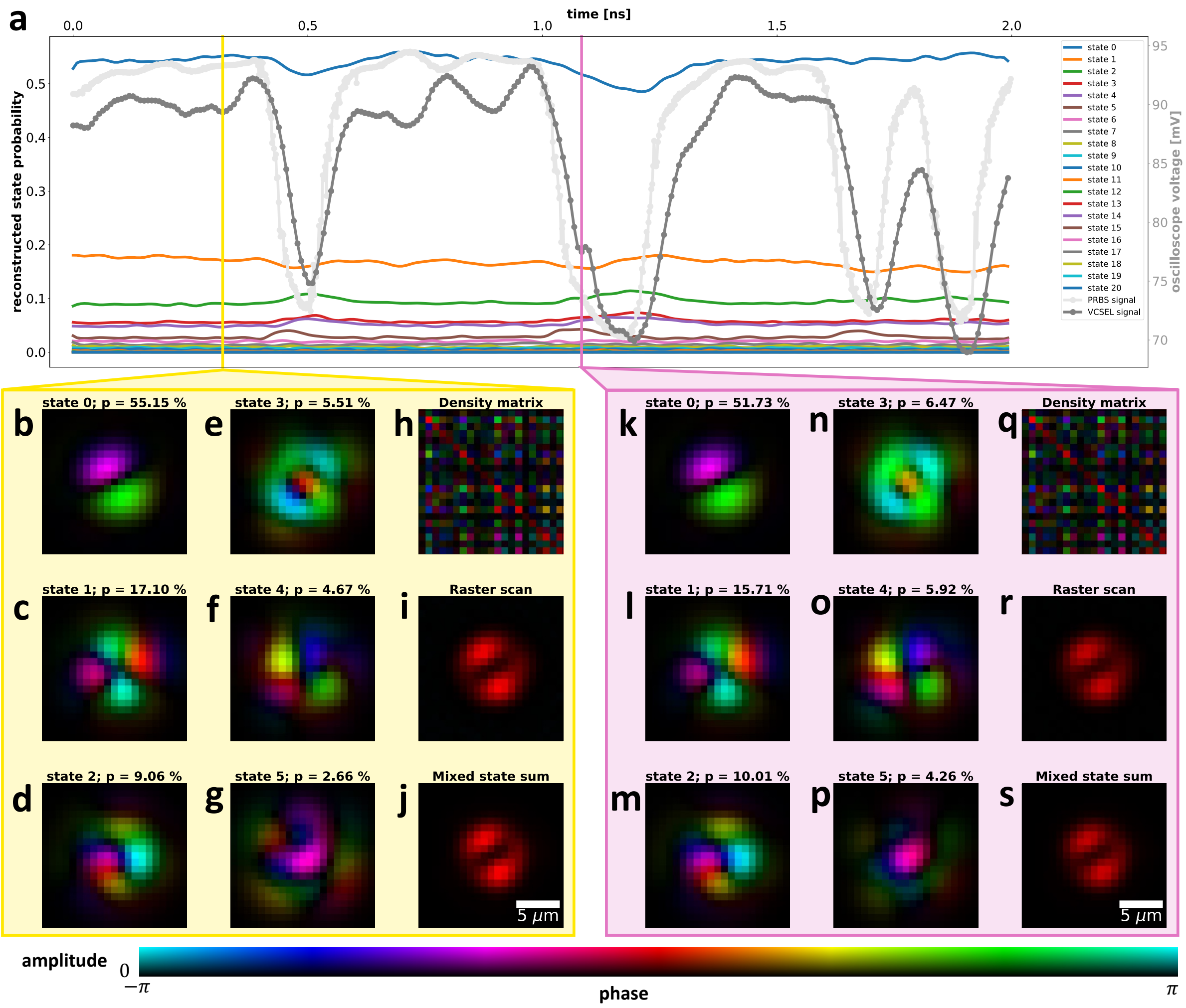

Supplementary Figure 4 **Spatio-temporal analysis of VCSEL (V polarisation)** (**a**) Probability of the reconstructed states (coloured lines) dynamically evolves during modulation (light-grey line) and differs from the overall VCSEL response (dark-grey line). (**b-g**) and (**k-p**) are the first six reconstructed spatial eigenstates of density matrices (**h**) and (**q**) acquired at times $t = 0.32$ ns and $t = 1.08$ ns respectively. (**i**) and (**r**) are the total spatial intensity profiles measured by the SLM raster-scan at times $t = 0.32$ ns and $t = 1.08$ ns respectively. (**j**) and (**s**) are the total spatial intensity profiles calculated as a probability-weighted sum of intensity profiles of all reconstructed spatial states at times $t = 0.32$ ns and $t = 1.08$ ns respectively. For the behaviour of the system over the whole 2 ns interval see Supplementary Media 4.

Supplementary Note 7: LIGHT-CURRENT CURVES OF INDIVIDUAL VCSEL MODES

The laser diode transverse (spatial) mode characteristics are driven by the applied bias current and the diode temperature. As a result, the modally resolved light-current (LI) curves unlock information that is necessary for understanding the VCSEL behaviour during modulation. Here, we study the light-current curves of the VCSEL at a fixed temperature of 68 C°. At low bias currents close to the threshold, only the fundamental mode is supported by the cavity as can be seen in Supplementary Figure 5 (a,c). This is due to the fundamental mode having tight

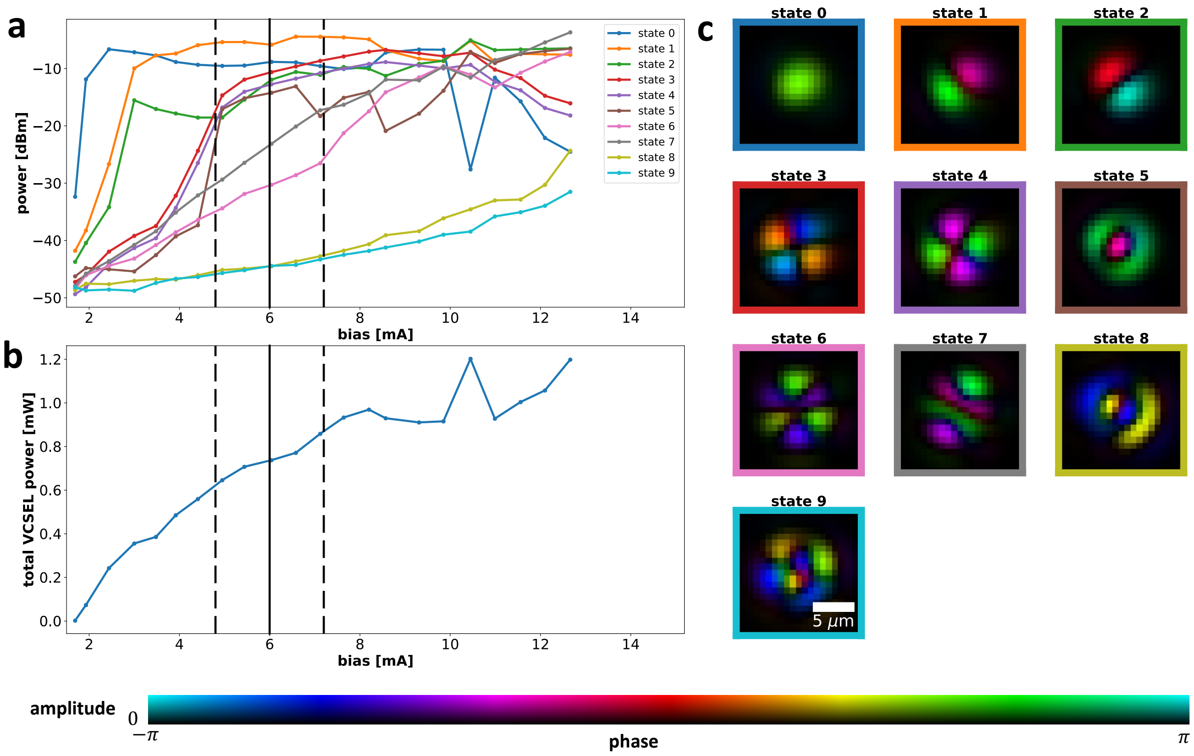

Supplementary Figure 5 **Light-current curves for individual modes (H polarisation)** (**a**) Modally resolved light-current curves with line colours matched to mode profiles in (**c**). (**b**) Total VCSEL power integrated over all the spatial modes as a function of bias current. (**c**) Mode profiles as detected by the high-dimensional Stokes analysis with frame colour-coded to correlate with line colours in (**a**). The vertical, solid, black line marks the bias current applied to the VCSEL in all static experiments in our manuscript. The two dashed vertical lines mark the bias current applied to VCSEL at the extremities of modulation during temporal dynamics experiments.

confinement in the cavity and as a result a lower loss coefficient. As the bias increases, the higher-order transverse modes with less confinement and higher loss coefficient start to be supported by the cavity and the relative power between the modes re-distributes in a way that favours higher-order modes.

During modulation experiments in our manuscript, we operate the VCSEL at an average bias current of 6 mA (marked with a solid black line in Supplementary Figure 5 (a), 6 (a)). The VCSEL at this bias supports 9 spatial modes (Supplementary Figure 5 (c), Supplementary Figure 6 (c)). The modally resolved light-current curves in Supplementary Figure 5 (a) and Supplementary Figure 6 (a) are obtained by repeating the high-dimensional Stokes analysis in Fig. 3 for multiple applied bias values in the range from 1.69 mA to 12.66 mA. For each bias value, we determine the power in the spectral peaks corresponding to a given spatial mode. In cases when the modes spectrally overlap, such as the case of LP21a and LP21b modes, we determine the total power in the spectral peak and use the Stokes state probabilities to retrieve the power of each spatial mode within the peak. We also measure the total power emitted by the VCSEL over the whole spectral range for each bias value, which yields the total light-current curve of the VCSEL for H polarisation (Supplementary Figure 5 (b)) and V polarisation (Supplementary Figure 6 (b)). The strong power peak observed at the bias value of 10.6 mA (Supplementary Figure 5 (b)) is cancelled out by a power dip for the same bias value in the V polarisation case (Supplementary Figure 6 (b)). This polarisation flip dynamics is due to the current injection pattern into the cavity. When the light-current curves for H and V polarisation (Supplementary Figure 7 (a)) are added together, we arrive to the typical light-current curve of laser diode (Supplementary Figure 7 (b)), with characteristic rollover at around 10 mA.

The dashed lines in Supplementary Figure 5 (a,b), 6 (a,b), 7 (a,b) mark the bias currents applied at respective extremes during VCSEL modulation, with results supporting the observed temporal dynamics behaviour in Figure 4 for H polarisation and Supplementary Figure 4 for V polarisation.

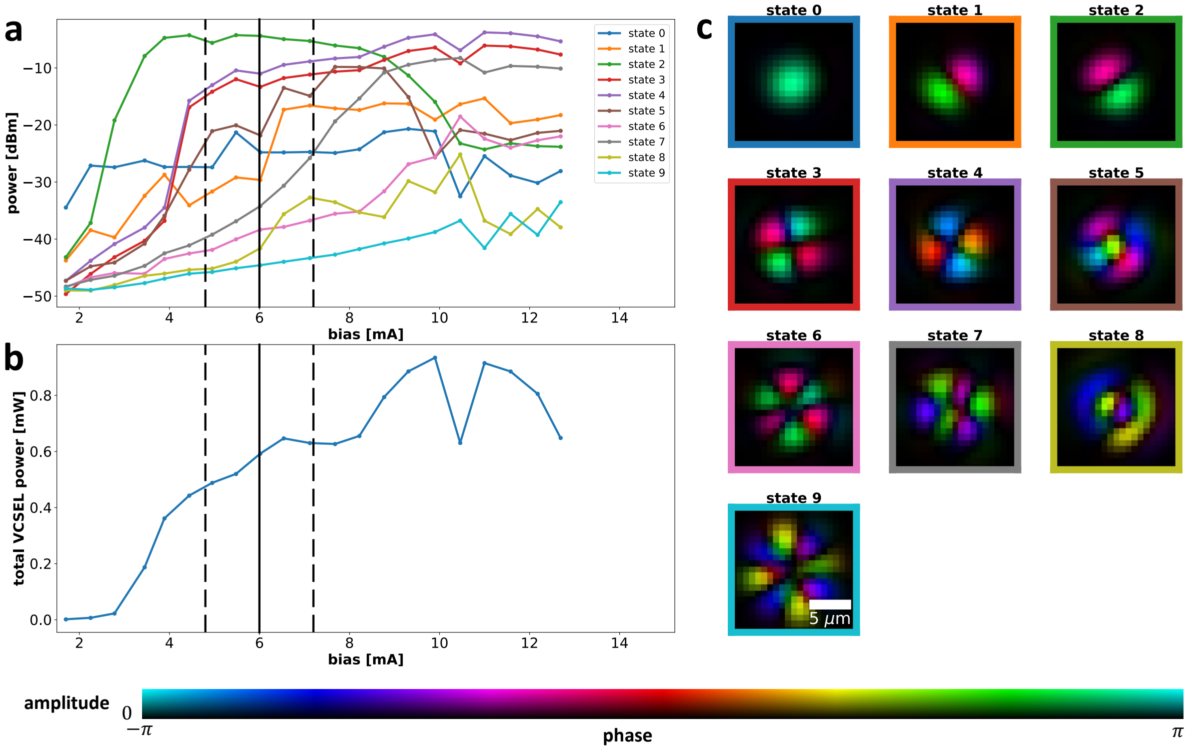

Supplementary Figure 6 **Light-current curves for individual modes (V polarisation)** (**a**) Modally resolved light-current curves with line colours matched to mode profiles in (**c**). (**b**) Total VCSEL power integrated over all the spatial modes as a function of bias current. (**c**) Mode profiles as detected by the high-dimensional Stokes analysis with frame colour-coded to correlate with line colours in (**a**). The vertical, solid, black line marks the bias current applied to the VCSEL in all static experiments in our manuscript. The two dashed vertical lines mark the bias current applied to VCSEL at the extremities of modulation during temporal dynamics experiments.

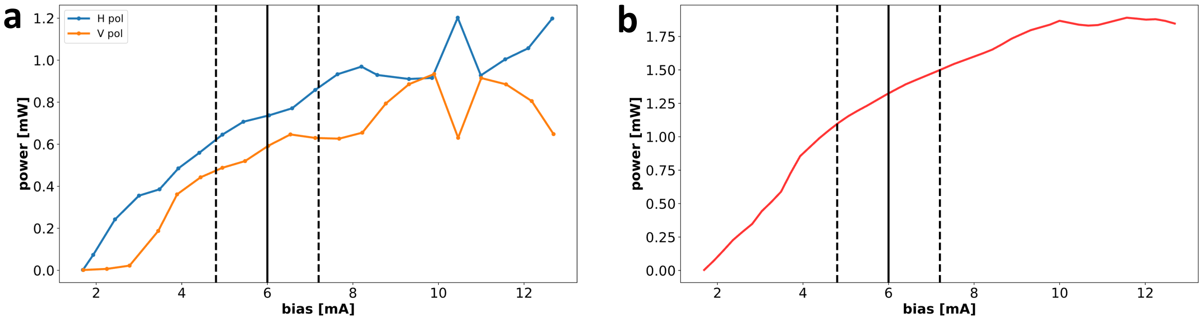

Supplementary Figure 7 **Polarisation resolved light-current curves** (**a**) Polarisation resolved light current curves for H and V polarisation. (**b**) The sum of H and V polarisation light-current curves yields the typical light-current curve of laser diode. The vertical, solid, black line marks the bias current applied to the VCSEL in all static experiments in our manuscript. The two dashed vertical lines mark the bias current applied to VCSEL at the extremities of modulation during temporal dynamics experiments.

Supplementary Note 8: RESOLVING SPECTRALLY OVERLAPPING SPATIAL MODES

One of the most powerful and intriguing abilities of the Stokes analysis is its ability to resolve several incoherent orthogonal spatial modes that are spectrally overlapping. This ability is enabled due to the density matrix containing information about the states and their probabilities in the mixture. If there are multiple spatial modes spectrally overlapping in a given wavelength bin, then these spatial modes form a mixture of states and the Stokes approach can resolve them. It is this property of the Stokes analysis that allows the determination of probabilities of the spectrally overlapping LP21a and LP21b spatial modes in Figure 3 in the main manuscript, and the spatially resolved analysis of the temporal dynamics in Figure 4, despite the oscilloscope having no spectral resolution. This can be very useful in situations when there is no access to a spectrometer, or when the spectrometer lacks the resolution needed to resolve the peaks. Moreover, the Stokes analysis is to our knowledge the only approach able to distinguish between multiple mutually incoherent modes versus a single state that would have the same intensity profile, *e.g.* two incoherent perpendicularly oriented LP11 versus a single LG01 which would have the same intensity profile. In the case of temporal analysis, the method can resolve individual modes arriving at the same delay which allows tracking of their dynamics in scenarios when the spectral filtering of spectrally tightly packed individual modes is very challenging or impossible with readily available spectral filters.

Here we verify the ability of the Stokes analysis to quantitatively determine the spatial states and their probabilities by taking the spectrum of the VCSEL with a low resolution bandwidth of the OSA, specifically 10 nm (Supplementary Figure 8, blue datapoints).

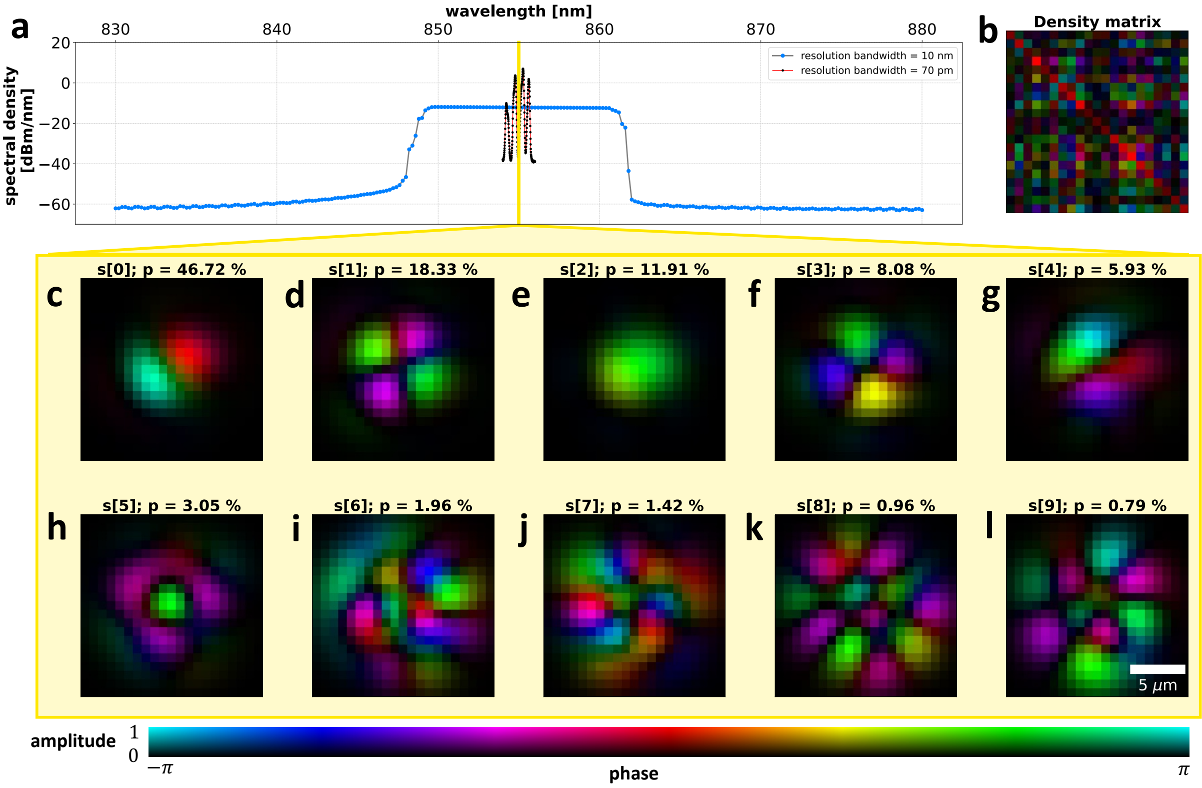

Supplementary Figure 8 **Resolving spectrally overlapping spatial modes** (**a**) VCSEL spectra with OSA resolution bandwidth set to 70 pm and 10 nm (**b**) The density matrix measured at $\lambda = 855$ nm with the resolution bandwidth of 10 nm. (**c-l**) First ten reconstructed spatial eigenstates of the density matrix (**b**).

The acquired spectrum does not resolve the individual spectral peaks, which are visible with the resolution bandwidth of the OSA set to 70 pm (Supplementary Figure 8, black data points). Despite the insufficient resolution to spectrally distinguish the modes in the low-resolution bandwidth scenario, when we apply the Stokes analysis to reconstruct the density matrix at $\lambda = 855$ nm (yellow vertical line), we do obtain the familiar spatial modes from Figure 3 and Figure 4 in the main manuscript. Not only do we reconstruct the correct spatial profiles but we also gain access to their probabilities.

The retrieved probabilities do have a physical meaning. We measured the power in the individual peaks from the high-resolution bandwidth spectrum (Supplementary Figure 8, black data points) and determined the total power in

the spectrum by summing the power of all the peaks. The ratio of peak powers and the total power then provides directly measured modal probabilities in the VCSEL beam (Supplementary Figure 9, blue bars). The directly measured probabilities are in very good agreement with the probabilities recovered from the Stokes analysis (Supplementary Figure 9, orange bars), which conclusively confirms that the Stokes analysis provides not only a qualitative insight into what spatial modes are present in the beam but it also provides a quantitative insight into the relative power of the spatial modes within the beam.

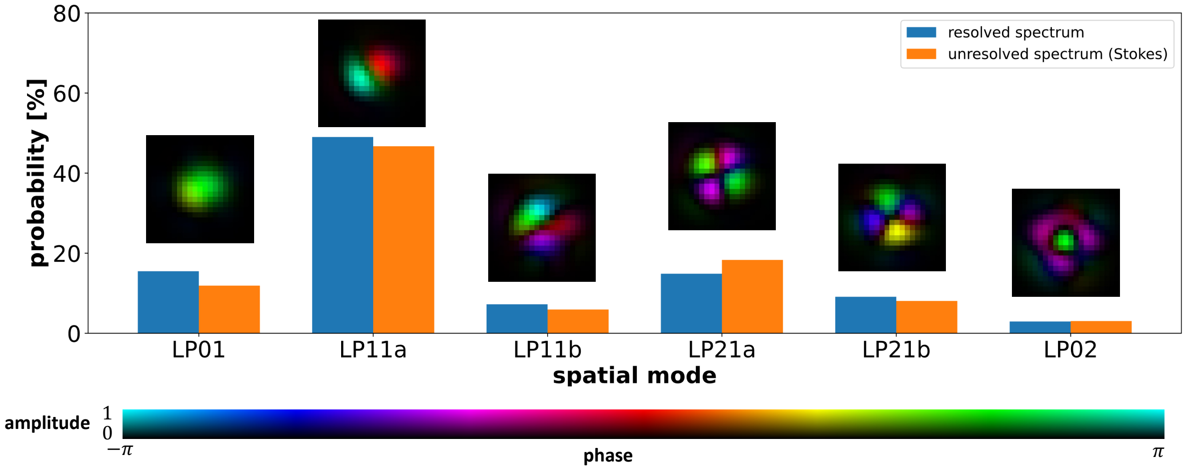

Supplementary Figure 9 **Stokes analysis for the quantitative determination of spatial mode probabilities in a mixture** The comparison of the retrieved modal probabilities in the optical beam recovered from the resolved spectrum (blue, resolution bandwidth of the OSA is 70 pm) and using the Stokes analysis for an unresolved spectrum (orange, a resolution bandwidth of the OSA is 10 nm). We note that for the case of LP21a and LP21b modes, the OSA is not able to spectrally resolve them even with a resolution bandwidth of 70 pm. The values of blue bars, in this case, are therefore determined by subdividing the power in the peak, containing both LP21a and LP21b, based on the probabilities acquired from the Stokes analysis in Figure 3, 62.98 % and 27.43 % respectively. This recursive usage of Stokes analysis is justified given the excellent match in probabilities for the resolved peaks.

Supplementary Note 9: SENSITIVITY OF THE HIGH-DIMENSIONAL STOKES ANALYSIS TO MEASUREMENT NOISE

The experimental noise is a complex issue in the high-dimensional Stokes analysis that depends not only on the level of power fluctuations of the source laser but also on the number of modes in the Stokes basis. Here we explore the effect of noise in several scenarios that are relevant to the experimental data presented in the main manuscript. In all the following numerical simulations, the noise values are generated from a normal distribution with a standard deviation σ equivalent to a specific percentage of the original unprojected signal average. For example, for 5% noise level, the standard deviation is $\sigma = 0.05\,\mu$, where μ is the signal average. Each projection acts on the original signal + normal distribution value, which affects the projected intensities, which in turn introduces the noise error into the reconstructed Stokes vector and finally into the corresponding density matrices. The resulting effect of added noise are density matrices that are not positive semidefinite and are therefore not physical. We apply the Higham[5] algorithm to find the nearest positive semidefinite matrix that makes physical sense - the larger the noise, the larger the typical distance in terms of Frobenius norm from the non-physical and the physical density matrix. The price we pay to obtain the physical matrix is the introduction of the noise induced error into the reconstructed probabilities and eigenstates. The above described numerical procedure closely follows the noise induced effect in a real measurement scenario.

In the following, we often want to assess the fidelity of the reconstructed wavefront with respect to the original wavefront. We use the normalised square of the overlap integral ($o = \left|\int E_1^* E_2 \,\mathrm{d}A\right|^2 / \left(\int |E_1|^2 \,\mathrm{d}A \int |E_2|^2 \,\mathrm{d}A\right)$; E_1 and E_2 are the fields to be overlapped) for this purpose, and refer to it throughout this section simply as overlap. In situations where the probability of the original wavefront is zero, we assume its normalisation factor equal to 1. This allows comparison of the original and reconstructed wavefronts in all scenarios of interest.

A. Spatio-spectral analysis; pure state scenario

As can be seen in Figure 3 of the main manuscript, the first two dominant spatial eigenstates typically have a combined probability of around 90%. Even in the case when the spectral peak contains only a single spatial eigenstate, such as the peak corresponding to the fundamental mode, the probability of the dominant spatial eigenstate is still only 90% and not 100% as expected for the pure mode. This discrepancy affects the fidelity of quantitative predictions that the high-dimensional Stokes method is capable of.

We identified the main culprit for the discrepancy to the power oscillation of the light source in the system. We have measured the variation of intensity of our light source for a fixed projective mask over a time of 30 minutes, which is a typical time needed to display and measure the 861 spatial analyser state for the Stokes analysis dimension of $N = 21$. The measured fluctuation in power was around 5%. We subsequently performed a numerical simulation of our spatio-spectral experiment for an input density matrix corresponding to a situation in which only the fundamental mode is present with a probability of 100% (Supplementary Figure 10 (a)), such as the fundamental peak in Figure 3 of the main manuscript. We then numerically calculated the expectation values for each projective mask and applied a 5% variation to the numerically acquired intensity measurements. Finally, the noise affected density matrix was cast into the positive, semi-definite form and its eigenvectors and eigenvalues were found. As evident in Supplementary Figure 10 (b) and (c) the reconstructed probability is around $90\% \pm 1.2\%$ (analysis of 20 different simulations with a random 5% noise) for the fundamental mode, with the remaining 20 Stokes eigenstates accounting for the remaining 10% (only 6 states are shown for clarity). The obtained probability is in excellent agreement to our experimentally measured spatio-spectral data. Interestingly, the fidelity of the eigenstate is not affected by the noisy measurement. The overlap of the input spatial state with the reconstructed spatial state is 0.9997. The fidelity of the reconstruction of the spatial state is maintained for even very high levels of noise as evident in Supplementary Figure 11 where we show the reconstruction for a 100% noise level. Despite the reconstructed probabilities being heavily affected by the noise level, the reconstructed spatial state overlap with the input state is still around 0.9.

In order to verify the trend of the relatively small effect of noise on the overlap, we numerically simulated the reconstructed probability in the dominant state and the overlap of the dominant eigenstate with the input state over a range of noise levels from 0% to 200% of the original signal (Supplementary Figure 12 (a,b)). As can be seen in Supplementary Figure 12 (b), the overlap is very stable with the increasing level of noise. We hypothesise that this is due to each eigenchannel being independent, and consequently, the noise is not additive. For the probability reconstruction, this is not true. As the noise increases, the probability of the $N - 1$ parasitic eigenchannels becomes non-negligible relatively fast, leading to an abrupt drop in the reconstructed probability of the dominant state as seen in Supplementary Figure 12 (a).

The reconstructed probabilities are also sensitive to the dimension of the Stokes analysis. The Supplementary Figure 13 shows the outcome of the numerical simulation with a 5% noise level, but for Stokes dimension of N = 55,

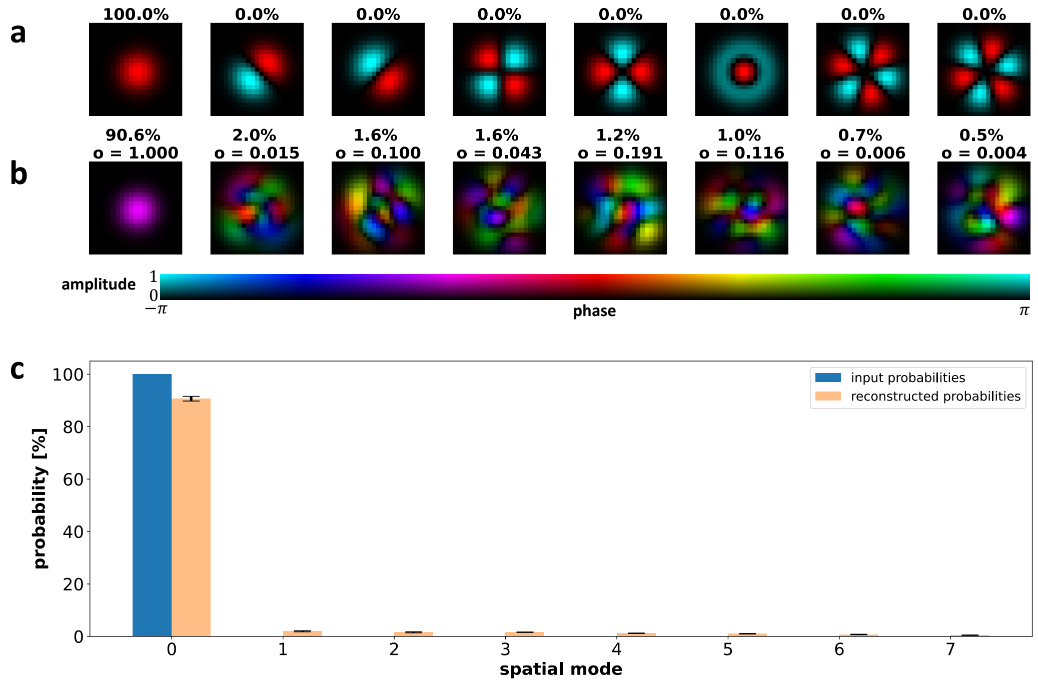

Supplementary Figure 10 **Stokes analysis for pure state and** 5% **noise** (**a**) The input is a pure state - a fundamental mode with probability of 100%. (**b**) The numerical simulation with a 5% standard deviation fluctuations of source power. Both the reconstructed probability and the overlap o of the reconstructed spatial mode with the input mode is highlighted. (**c**) The input and the reconstructed probabilities over the first 8 modes with error bars showing the standard deviation over 20 different numerical simulations.

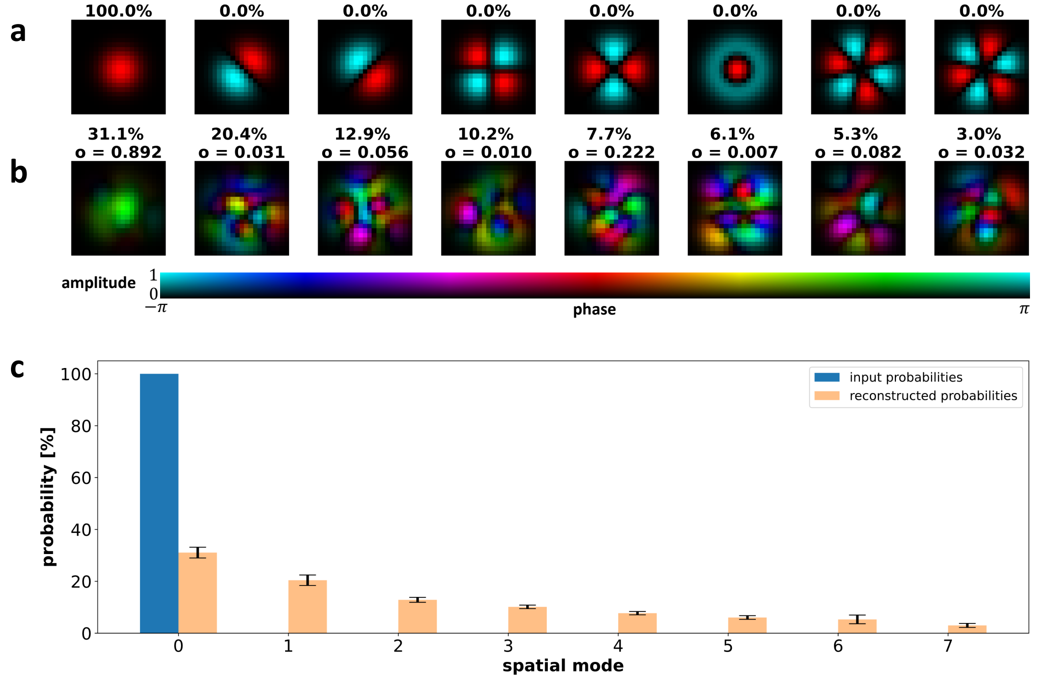

Supplementary Figure 11 **Stokes analysis for a pure state and a** 100% **noise level** (**a**) The input is a pure state - a fundamental mode with probability of 100%. (**b**) The numerical simulation with a 100% noise level. Both the reconstructed probability and the overlap o of the reconstructed spatial mode with the input mode are highlighted. (**c**) The input and the reconstructed probabilities over the first 8 modes with error bars showing the standard deviation over 20 different numerical simulations.

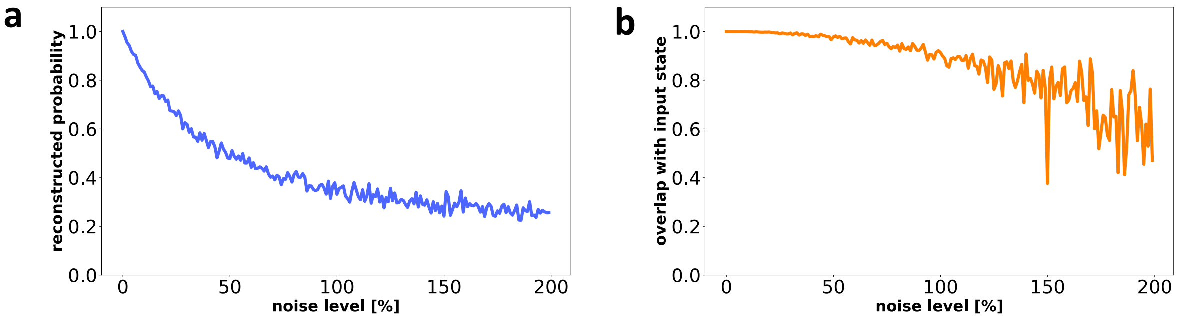

Supplementary Figure 12 **Effect of measurement noise on Stokes analysis** (**a**) The reconstructed probability of the dominant eigenstate as a function of measurement noise. (**b**) The overlap of the reconstructed dominant spatial eigenstate with the input state as a function of measurement noise. The amplitude of the reconstructed spatial profile is normalised to one for overlap calculations.

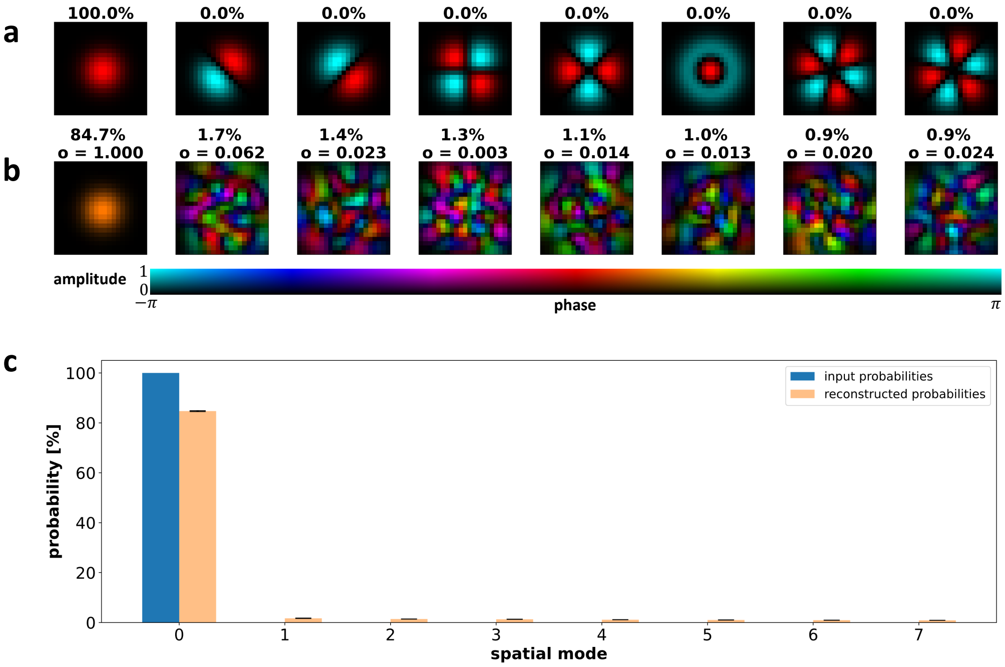

Supplementary Figure 13 **Stokes analysis for pure state,** 5% **noise level and Stokes dimension of** $N = 55$ (**a**) The input is a pure state - a fundamental mode with probability of 100%. (**b**) The numerical simulation with a 5% noise level fluctuations of source power. Both the reconstructed probability and the overlap o of the reconstructed spatial mode with the input mode is highlighted. (**c**) The input and the reconstructed probabilities over the first 8 modes with error bars showing the standard deviation over 20 different numerical simulations.

which requires 5995 state analyser states. The overlap of the reconstructed spatial state with the input state is still excellent and close to 1, whereas the reconstructed probability of the dominant state drops to around 85%. We note that the reconstructed probability shows minimal changes in the 20 numerical simulations as evident from the error bars in the Supplementary Figure 13 (c).

The effect of noise on probability reconstruction fidelity of the dominant mode is lower when we decrease the dimension of the Stokes analysis to $N = 6$ which requires a total of 66 projective measurements (Supplementary Figure 14).

In brief, the higher the dimension of the Stokes analysis, the worse is the probability reconstruction, likely due to the noise feeding into more and more eigenstates of the density matrix. These parasitic eigenstates have a small non-zero probability due to the noise and when added over $N - 1$ eigenstates, they significantly affect the dominant state's probability value. However, the effect of noise on the reconstructed spatial eigenstates seems to be negligible. This is likely due to a non-cumulative effect of the noise on eigenvectors of the density matrix.

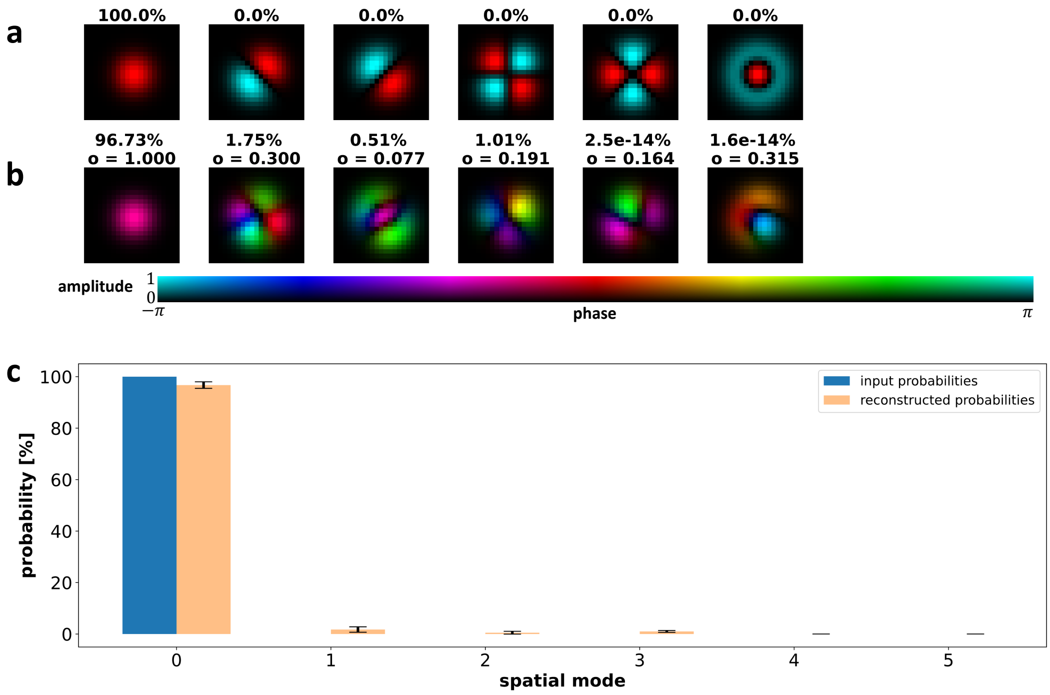

Supplementary Figure 14 **Stokes analysis for pure state,** 5% **noise level and Stokes dimension of** $N = 6$ (**a**) The input is a pure state - a fundamental mode with probability of 100%. (**b**) The numerical simulation with a 5% noise level fluctuations of source power. Both the reconstructed probability and the overlap o of the reconstructed spatial mode with the input mode is highlighted. (**c**) The input and the reconstructed probabilities over the first 6 modes with error bars showing the standard deviation over 20 different numerical simulations.

B. Spatio-temporal analysis; mixed state

In the case of the spatio-spectral analysis, there is typically only one or two spatial modes in any wavelength bin and we explored the effect of noise for this pure state case in detail above. This contrasts with the spatio-temporal analysis case, with the Stokes analysis dealing with all the spatial states in each temporal bin. Here we explore the effect of noise in this mixed state scenario. We perform a numerical simulation with the input probabilities of states in the mixture proportional to the experimental power measured for each spatial state, as acquired from spectra in Figure 3 of the main manuscript. These are the same probabilities as in Supplementary Figure 9 and represent the typical power distribution between modes for most of the results presented in the manuscript.

Supplementary Figures 15 (a,b) show the input states and their reconstruction with no noise in the system. In the mixture, there is no defined phase relationship between the incoherent states and also no way of defining absolute phase overall. As a result, there is the random phase difference and also an undefined absolute phase between the input states and the reconstructed states. Otherwise, the reconstruction of the probabilities and the corresponding spatial eigenstate wavefronts is perfect. Increasing the noise level to 1% (Supplementary Figure 15 (c)) affects only the reconstruction of the weakest state in the mixture as expected. The interesting behaviour is observed for the noise level of 3% (Supplementary Figure 15 (d)). The two weakest spatial states (LP31a and LP31b) are most strongly affected by noise as expected. But interestingly, there is a very significant effect on LP01 and LP21a spatial states, even though they have relatively high input probabilities in the mixture of 15.5% and 15.0% respectively. Their overlap with the input spatial states drops to 0.92 and the shape of the fundamental LP01 mode is visibly affected, similar to what we have observed experimentally in Figure 4 of the main manuscript. This stems from the fundamental inability to resolve spatial states with the same probability (power) in the mixture. For example, there is no way to distinguish whether a completely unpolarised light was generated by combining two vertically and horizontally polarised sources together, or two left and right hand circularly polarised. It is not even a meaningful question to ask, since the mixture is completely random, with no preferred basis, even from the perspective of the physical process generating the mixture. From the mathematical point of view, in the case of the two dimensional Stokes and 50/50% mixture, we end up in the center of the Bloch sphere, where we cannot make any conclusion about the spatial states of the mixture. Each of the states can be any linear combination of the two basis states. Theoretically, without noise, such uncertainty only appears in the center of the Bloch sphere, but in the presence of noise the uncertainty region is

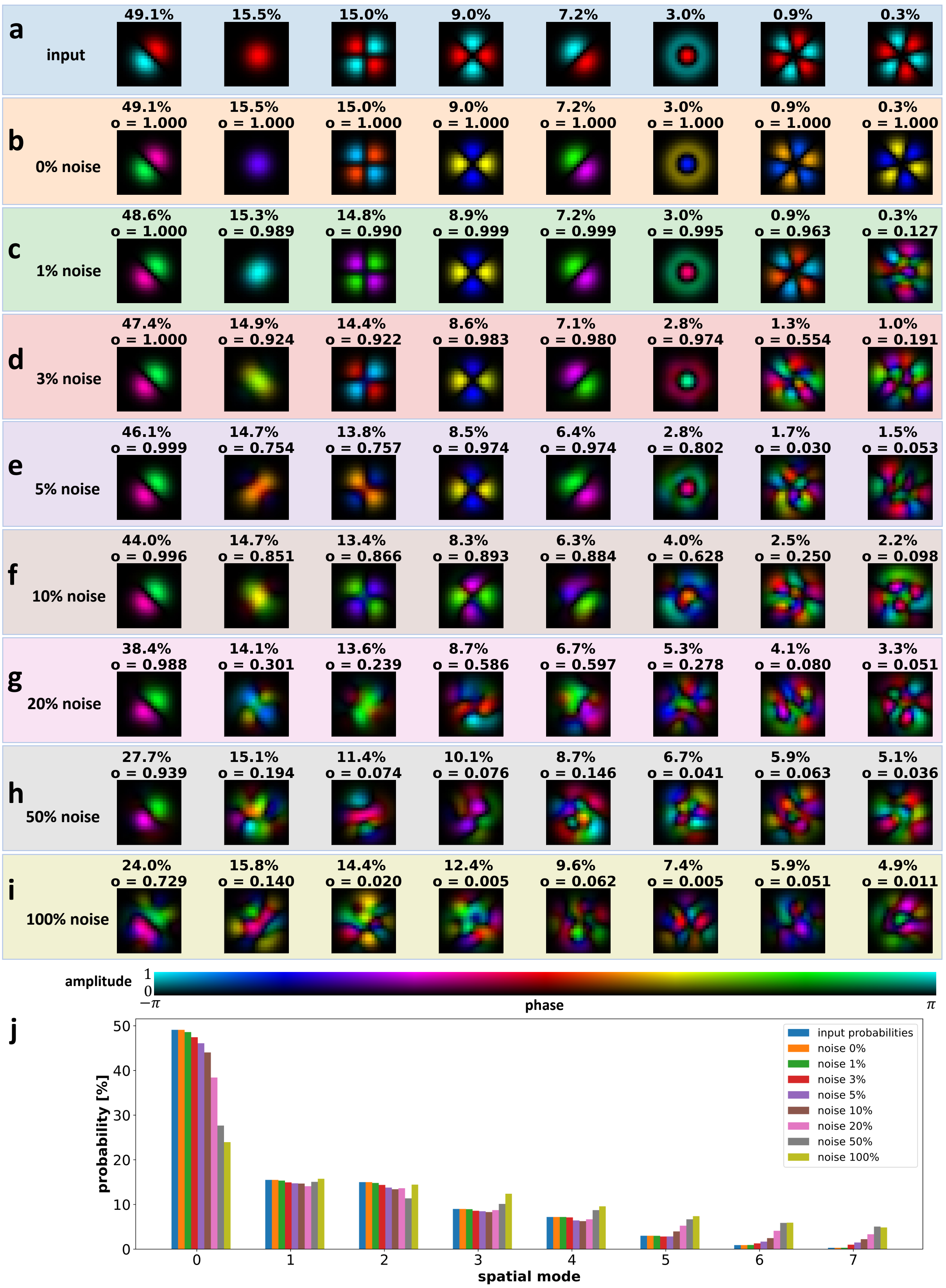

Supplementary Figure 15 **Stokes analysis for mixed state with varying levels of noise** (**a**) The input mixed state's spatial states and probabilities (**b-i**) The reconstructed spatial eigenstates, their probabilities and overlaps (o), between the input and the reconstructed eigenstate, numerically simulated for varying level of signal noise. (**j**) The reconstructed probability for the first 8 reconstructed eigenstates for varying level of noise. The numerical simulation was performed for Stokes dimension of $N = 21$.

not just a center of the Bloch sphere but a small sphere around its origin, with the diameter directly proportional to the noise level. Since the LP01 and LP21a have very similar input probabilities of 15.5% and 15.0% they end up in one of the uncertainty regions of the Bloch hyperball and their expected spatial profile starts to be a linear combination of the two input states. With the increasing noise, this effect becomes stronger as can be seen in (Supplementary Figure 15 (e-f)), where the much weaker LP21b, LP11b and LP02 can still be resolved, but the stronger LP01 and LP21a are strongly affected. Further increase of the noise level as in Supplementary Figure 15 (g-i) ultimately affects all the input states and their reconstructed probabilities. The summary of the reconstruction fidelity is depicted on Supplementary Figure 15 (j).

The worst possible scenario for the Stokes analysis is the case when all input probabilities are exactly equal (completely mixed state). Such states cannot be distinguished even in the absence of noise. The representation of such a mixed state is at the origin of the Bloch hyperball, this time with each state possibly being a linear combination of all the states in the mix. The Supplementary Figures 16 (a,b) show the reconstruction of the probabilities and

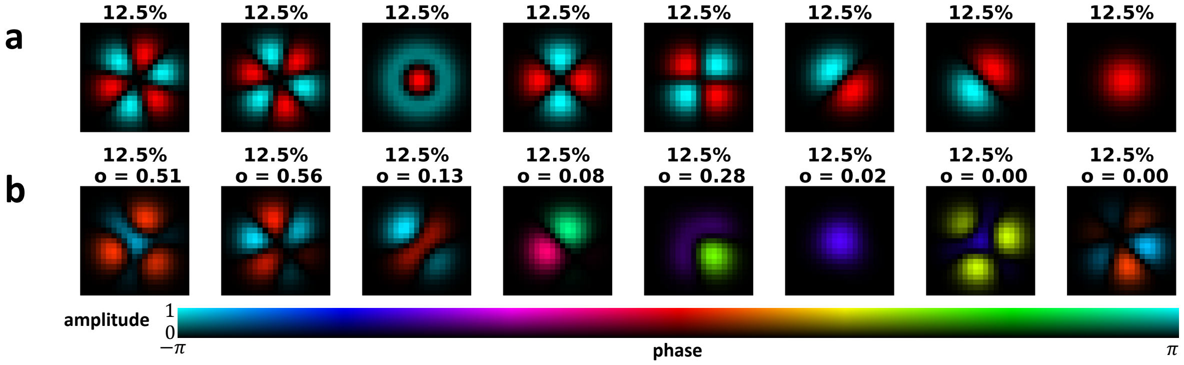

Supplementary Figure 16 **Stokes analysis for a completely mixed state** (**a**) The input mixed state's spatial states and probabilities (**b**) The reconstructed spatial eigenstates and their probabilities. The numerical simulation was performed for Stokes dimension of $N = 21$.

the reconstructed eigenstates for such a completely mixed state, with Stokes analysis failing to determine the states in the mixture correctly. Even though the dimension of the Stokes analysis is $N = 21$, the reconstructed states are only a linear combination of the 8 mixture states. If the input mixture had 21 completely mixed states, then the reconstructed wavefronts would be a linear combination of all the input states.

At this point, it is also interesting to explore a case when the mixture is made of input states with a similar but not exactly the same probability. This is experimentally a much more likely scenario than all the input states having exactly the same probability. The results of such numerical simulation are presented in Supplementary Figure 17. The Stokes analysis can resolve all the states in the mixture when there is no noise in the system. Even at low levels of noise around 1%, most of the spatial states in the mixture are still recognisable. At a noise level of 3%, the input spatial states can no longer be resolved, with each wavefront being a linear combination of input states in the mixture. These numerical results show that the Stokes analysis is capable of reconstructing the states in the mixture even in scenarios when the input is almost completely mixed.

C. Orthogonality

The noise effect on the reconstructed spatial eigenstates is only one source of error in measurements such as the spatio-temporal analysis presented in Figure 4 of the main manuscript. Another source of wavefront distortion is due to not perfectly orthogonal modes of the laser cavity. For the analysis to work, the input mixture has to be made of spatially orthogonal states. If the states in the mixture are not orthogonal, the density matrix is no longer a unique representation of the mixture. This is not a problem of the Stokes approach itself, but a deeper, fundamental inability to distinguish the non-orthogonal spatial states based purely on spatial measurement. For example, similar situation would arise in the spectral domain. If there are two sources at exactly the same wavelength (non-orthogonal), it is fundamentally not possible to distinguish between them spectrally – the only way is to try and differentiate them either in the time or the spatial domain.

The effect of non-orthogonal input states is demonstrated in Supplementary Figure 18, where we rotate the odd and even LP11, LP21, and LP31 modes by 3° (experimentally observed from the SLM raster scan) with respect to their orthogonal orientations (Supplementary Figure 18 (a)). The reconstructed states and their probabilities are presented in Supplementary Figure 18 (b). The most noticeable effect is on the LP21 odd and even modes, with the

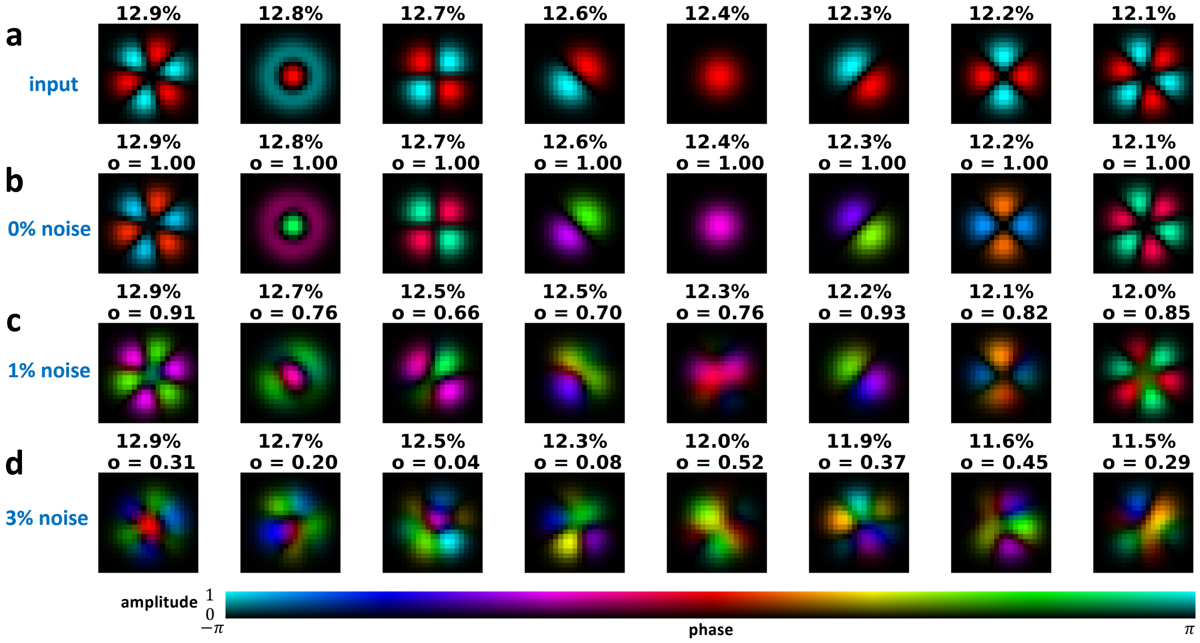

Supplementary Figure 17 **Stokes analysis for an almost completely mixed state** (**a**) The input mixed state's spatial states and probabilities (**b-d**) The reconstructed spatial eigenstates and their probabilities for increasing levels of noise. The numerical simulation was performed for Stokes dimension of $N = 21$.

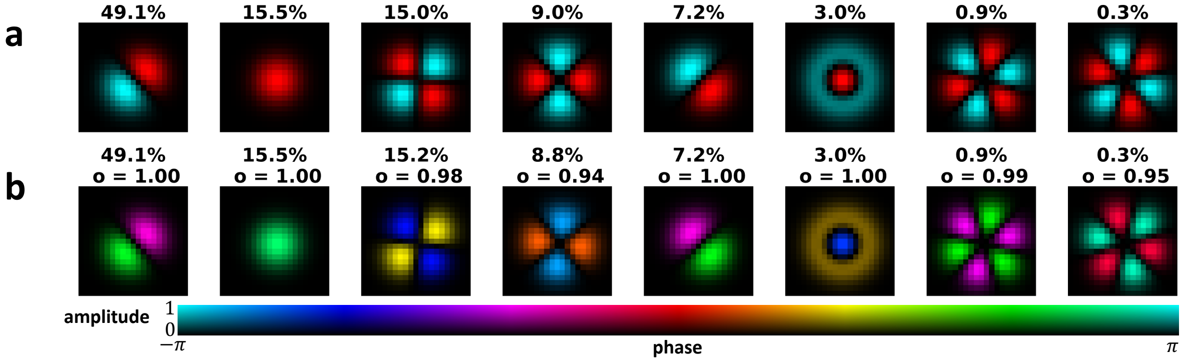

Supplementary Figure 18 **The effect of orthogonality of input states on reconstruction** (**a**) The input mixed state's spatial states and probabilities with odd and even LP11, LP21 and LP31 modes rotated by 3° with respect to their orthogonal orientation. (**b**) The reconstructed spatial eigenstates and their probabilities. The numerical simulation was performed for Stokes dimension of $N = 21$.

detected orientation slightly different from the input and the reconstructed probabilities differing by 0.2% from the probabilities of the input states. We can therefore conclude that in the studied case, the lack of perfect orthogonality has negligible effects on the measurement. However, the orthogonality of input states has to be carefully considered when using the Stokes analysis in a general beam profiling scenario.

Supplementary Note 10: DESIGN, PERFORMANCE AND APPLICATION OF SPATIAL FILTERS

While studying the system dynamics as a whole has its advantages, the presence of noise and its effect on the spatial fidelity of reconstructed modes can be detrimental, especially when the system supports many modes that have to be unmixed simultaneously. Additionally, in some scenarios, only one spatial field within the beam may carry information of interest, with the rest only contributing to noise. In such scenarios, it might be beneficial to filter out the spatial modes of interest one by one and study them in isolation. We have already designed SLM masks that filter out, or project, only certain spatial profiles – the spatial analyser state filters. However, these were generated to facilitate Stokes method in RLG basis, with no *a priori* knowledge of the spatial mode composition of the beam. After performing the Stokes analysis, we can take advantage of the exact knowledge of spatial modes in the source, including not only the exact waist but also any minor deviations from perfect mode profile due to the real-world cavity deformation, to directly create SLM filters that are only sensitive to the existing spatial modes in the beam[8]. We calculate the spatial mode filters from the reconstructed modes obtained in spatio-spectral analysis (Fig. 3(c-e)) via the same method used for calculation of spatial analyser states (more information in Supplementary Note 4).

The modes selected for filtering (column in Supplementary Fig. 19(a)) are filtered by SLM masks (column in Supplementary Fig. 19(b)) and coupled into an SMF in the same way as during the Stokes measurement. The column of Supplementary Fig. 19(c) shows the initial spectra of the input light with no filter applied (light-grey, power in each wavelength bin integrated over all the spatial pixels of the SLM raster scan) and the spectra with the filter applied and the mask efficiency factored in dark blue. The light-blue curve then shows the raw filtered spectra with visible overall loss due to filter mask efficiency. Each figure in the column of Supplementary Fig. 19(c) also contains the extinction ratio value of a selected mode with respect to the power in all the other modes. The filters are capable of picking off individual spectral peaks even in cases when the other modes dominate in the unfiltered case, such as LP11 being 4 dB stronger than LP01 without the filter but 7 dB weaker after the application of the filter. On average, the filter increases the contrast for the selected mode by more than 10 dB. Interestingly, the spatial filter can resolve between spectrally overlapping but spatially orthogonal LP21a and LP21b modes, as evidenced by spectral peak offset between the two applied filters. This is an indispensable attribute in scenarios when spectral filtering is not physically feasible due to close proximity of spectral peaks. For modes that are weak *ab inicio*, the filter still improves their contrast. However, the more powerful modes prevent sufficiently high contrast to study these modes in isolation unless a narrow spectral filter is applied.

Finally, the column in Supplementary Fig. 19(d) shows the oscilloscope traces for the filtered modes (black datapoints) with markedly different temporal behaviour, which closely follows the expected behaviour based on the modally resolved light-current curves as presented in Supplementary Note 7. For the spatial filters with a high extinction ratio value, these traces predominantly reflect the individual temporal behaviour of filtered modes with the contribution from other modes given by the extinction ratio of the filter. The sum of all filtered temporal traces (light-red curve, efficiency of the filter mask factored in) matches well the integrated temporal response measured by SLM raster-scanning (dark-red curve). Such a match can only be observed if there is minimal leakage of signal from other modes into the filtered mode which independently verifies that the SLM filters work as expected. The small discrepancy between the raster scan curve and the sum of all filtered modes is likely due to the suboptimal filtering of spatial filters with low extinction ratio, mainly for the LP01 and the LP21b filter case, which have non-negligible power and −6.98 dB and −4.60 dB extinction ratio respectively.

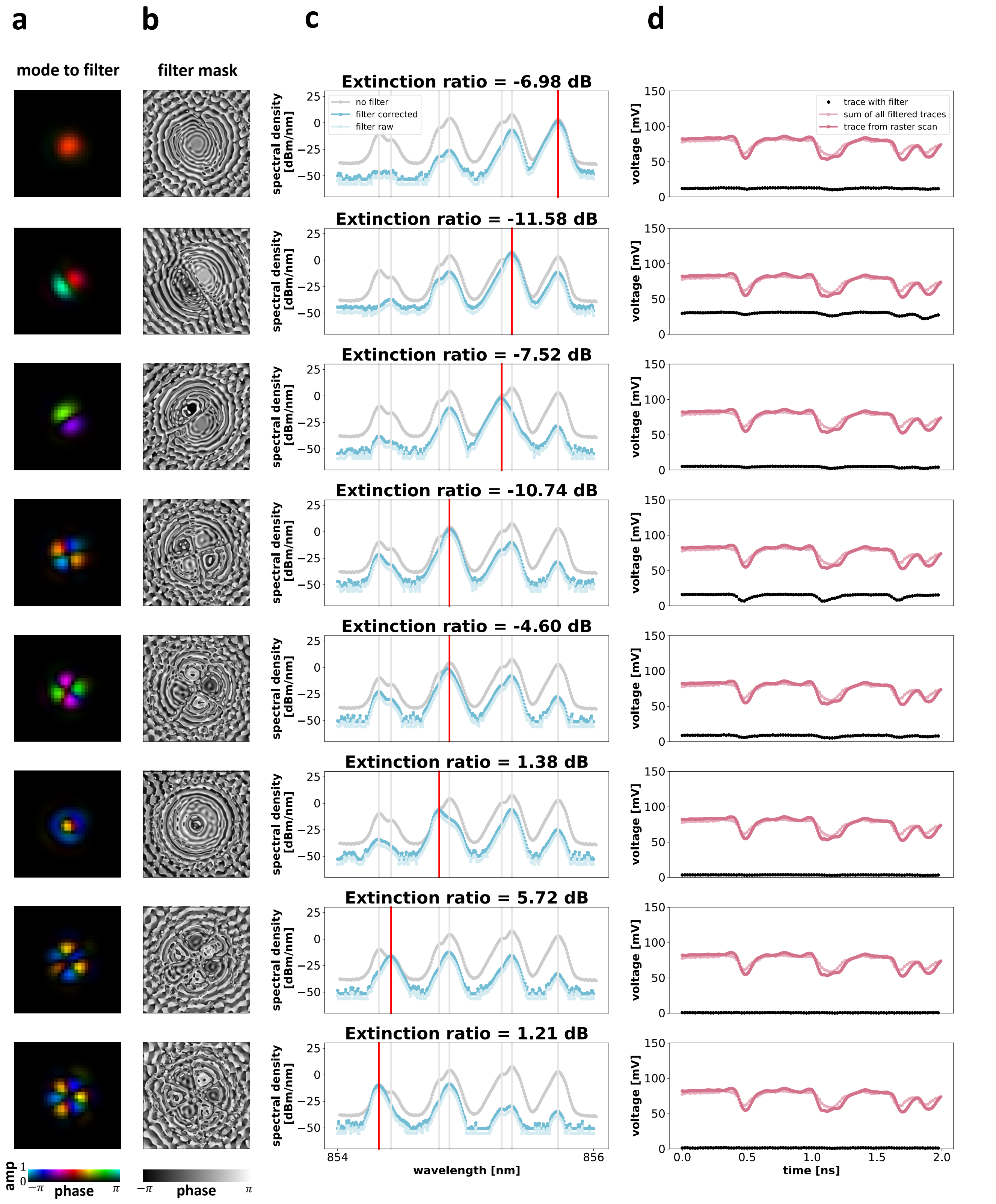

Supplementary Figure 19 **Spatial filters for spectral and temporal filtering of individual spatial states** (**a**) Modes selected for filtering as measured in the spatio-spectral analysis. (**b**) SLM masks filtering the corresponding modes in (**a**). (**c**) Performance of the filters based on comparison of unfiltered total power spectrum (light-grey) and the filtered spectrum corrected for loss of the filter mask (dark blue) and the raw filtered spectrum (light blue). The extinction ratio between the selected filtered mode and all remaining modes marked for each case. (**d**) Temporal traces taken with the applied spatial filters (black datapoints) along with the integrated temporal response obtained from the SLM raster scan (dark-red) and the sum of all the filtered traces (light-red). The acquired temporal signal was corrected for the efficiency of filter masks to facilitate the comparison with the integrated raster scan response.

References

1. Toninelli, E. *et al.* Concepts in quantum state tomography and classical implementation with intense light: a tutorial. *Adv. Opt. Photon.* **11**, 67 (2019).
2. Gell-Mann, M. Symmetries of Baryons and Mesons. *Phys. Rev.* **125**, 1067–1084 (1962).
3. Carpenter, J. & Fontaine, N. K. Optical single-shot spatial state tomography. In *14th Pacific Rim Conference on Lasers and Electro-Optics (CLEO PR 2020)*, C10C_4 (OSA, Sydney, 2020).
4. Fontaine, N. K. *et al.* Laguerre-Gaussian mode sorter. *Nat Commun* **10**, 1865 (2019).
5. Higham, N. J. Computing a nearest symmetric positive semidefinite matrix. *Linear Algebra and its Applications* **103**, 103–118 (1988).
6. Plöschner, M. & Čižmár, T. Compact multimode fiber beam-shaping system based on GPU accelerated digital holography. *Opt. Lett.* **40**, 197 (2015).
7. Bouchard, F. *et al.* Measuring azimuthal and radial modes of photons. *Opt. Express* **26**, 31925 (2018).
8. Carpenter, J., Thomsen, B. C. & Wilkinson, T. D. Degenerate Mode-Group Division Multiplexing. *J. Lightwave Technol.* **30**, 3946–3952 (2012).
