# Supplementary material for: Spatial tomography of light resolved in time, spectrum, and polarisation
Source: Nat Commun. 2022 Jul 25;13:4294. doi: 10.1038/s41467-022-31814-2 (PMC9314355; doi:10.1038/s41467-022-31814-2)
Supplement: Supplementary file 2 — Description of Additional Supplementary Files [file 41467_2022_31814_MOESM2_ESM.pdf]

Description of Additional Supplementary Files

File Name: Supplementary Movie 1
Description: Spatio-spectral analysis of VCSEL (H polarisation)

File Name: Supplementary Movie 2
Description: Spatio-spectral analysis of VCSEL (V polarisation)

File Name: Supplementary Movie 3
Description: Spatio-temporal analysis of VCSEL (H polarisation)

File Name: Supplementary Movie 4
Description: Spatio-temporal analysis of VCSEL (V polarisation)
